# Regulation of iron homeostasis by Fur and atypical response regulator SsoR via derepressor-inhibitor oscillation in *Shewanella oneidensis*

**DOI:** 10.1128/aem.01230-25

**Published:** 2025-08-27

**Authors:** Kaiyue Jie, Xinyue Liu, Jiyuan Hou, Peilu Xie, Jiaxin Tang, Haichun Gao

**Affiliations:** 1 Institute of Microbiology, State Key Laboratory for Vegetation Structure, Function and Construction (VegLab), College of Life Sciences, Zhejiang University 12377, Hangzhou, China; Michigan State University, East Lansing, Michigan, USA

**Keywords:** iron homeostasis, transcriptional regulation, phosphorylation-independent response regulator, Fur, activator-inhibitor oscillation

## Abstract

Iron is a vital cofactor for enzymes essential to many biological processes, yet in excess, it poses a danger to all living organisms. In order to ensure survival and proliferation under fluctuating environmental iron levels, bacteria evolve sophisticated regulatory systems to maintain iron homeostasis. Unlike master regulator Fur, a large portion of other players remains poorly defined. Here, we characterized the physiological impacts of atypical phosphorylation-independent response regulator SsoR of *Shewanella oneidensis*, a γ-proteobacterium renowned for metabolic versatility. By combining transcriptomics, proteomics, and transposon screening, we discovered that the SsoR loss impairs growth and decreases cytochrome *c* content under iron-limited conditions. Further investigations revealed that the defects can be attributed to lowered heme and iron levels, a consequence of elevated Fur production. Together, our findings suggest that SsoR and Fur constitute a derepressing-inhibiting oscillation system in maintaining iron homeostasis, providing a new composite view of regulator dynamics during the regulation of iron homeostasis in bacteria.

**IMPORTANCE:**

*Shewanella* comprises a large group of bacteria that are ubiquitous, ecologically widespread, and metabolically versatile, having enormous potential in biotechnology, environmental remediation, and energy production. These characteristics and applications are crucially determined by a myriad of iron-containing proteins, whose activity depends on the intricate regulation of iron homeostasis. Our study reveals that a derepressing-inhibiting oscillation system composed of Fur and atypical phosphorylation-independent response regulator SsoR plays a key role in the regulation of iron homeostasis at the transcription level. The loss of either results in altered production of the other, leading to disruption of iron homeostasis, which is harmful to the cell, especially under iron-limited conditions. This study deepens our understanding of the interacting dynamics of multiple regulators in iron homeostasis.

## INTRODUCTION

Iron primarily acts as a cofactor of various proteins required for diverse biological processes, such as respiration, DNA synthesis, and metabolism, and thus is an essential element for the survival and proliferation of almost all living organisms on Earth (1). In the meantime, excess iron is highly toxic to the cell by leading to oxidative stress, which damages chromosomes, lipid membranes, and redox proteins (2). As a result, iron homeostasis involving uptake, storage, utilization, and export is tightly regulated by an intricate network consisting of a variety of regulators that function at multiple levels through sophisticated mechanisms (1–3).

At the transcription level, many regulators that sense intracellular iron concentrations and modulate gene expression have been known, among which Fur (ferrous uptake regulator) is widely distributed and most extensively studied (4). After transcription, a number of small RNAs, RhyB in particular, along with RNA chaperones such as Hfq, affect expression by base-pairing with target mRNAs or interacting with proteins, influencing translation, stability, and degradation of transcripts (5). Moreover, given that a large portion of iron- and heme-containing proteins require a post-translational biogenesis process to be active, regulation after translation is also crucial, for example, IscR regulating Fe-S biogenesis in *Escherichia coli* (6–8).

In the *E. coli* paradigm, Fur acts as a master regulator in iron homeostasis by repressing gene transcription using ferrous iron (Fe^2+^) as a co-repressor (3). Under iron-repletion conditions, Fur interacts with Fe^2+^ to form Fur-Fe^2+^ complexes, which bind to *cis*-acting regulatory sequences known as iron-responsive elements (Fur boxes) to block transcription of operons that follow. When iron is in shortage, Fur loses the metal co-repressor and becomes inactive, leading to derepression (3). Despite this, Fur may function through other regulatory modes, including transcriptional activation by the metalated regulator and repression by the apo-protein (3, 9). In the case of apo-Fur, the DNA-binding motifs (apo-Fur boxes) differ from the consensus sequence of the classical Fur box (10, 11). In addition, Fur regulation can be further complicated by interactions with other regulators, in either a competitive or cooperative manner, and even with other iron-proteins for sensing intracellular iron homeostasis (3, 12–14).

Recently, we illustrated a previously undescribed mechanism for SsoR, a unique two-component system (TCS) DNA-binding response regulator that modulates siderophore production in *Shewanella*, which comprise a large group of γ-proteobacteria renowned for remarkable respiratory versatility and thus hold significant promise in bioenergy, including applications in microbial fuel cells, bioelectrochemical systems, and biohydrogen production (15–17). The respiratory versatility of *Shewanella* is in large part due to a vast number of iron-containing proteins, especially hemoproteins such as cytochrome (cyt) *c* proteins, and therefore, these bacteria have an iron demand substantially higher than model organisms, such as *E. coli*, requiring particularly tight regulation of iron homeostasis (18, 19). SsoR is conserved in *Shewanella* and closely related genera, and in *Shewanella oneidensis*, the research model for the genus, Fur senses iron availability and represses transcription of the *ssoR* gene (15). The uniqueness of SsoR is that SsoR is a genuine orphan TCS response regulator functioning in a phosphorylation-independent manner as an activator for transcription of the siderophore biosynthesis operon (15). Unlike other types of phosphorylation-independent TCS response regulators, which could not be phosphorylated due to the loss of the residue for phosphorylation, SsoR retains the ability to be phosphorylated but is structurally locked in the activation status (15, 20). This means that once produced, SsoR is active, and its activity increases with the amount of the protein. If unchecked, as in the absence of Fur, it would eventually lead to the disruption of iron homeostasis. Thus, the cell must be able to prevent its overproduction.

In this study, we made attempts to unveil the mechanism through which *S. oneidensis* keeps SsoR within safe levels. By examining the physiology impacts of SsoR, we found that the SsoR loss exhibits slow growth and lowered cyt *c* content under iron-deficient conditions. These phenotypes are not attributed to disruptions in siderophore synthesis but are instead linked to elevated Fur levels, as revealed through a combination of proteomics, transcriptomics, and transposon mutagenesis screening. Further analyses demonstrated that the phenotypes are associated with dysregulation in heme homeostasis and are ultimately caused by the reduction in both total and labile iron levels. All of these findings suggest that SsoR and Fur constitute a transcriptional derepressor-inhibitor oscillation system to regulate iron homeostasis. This work not only adds an additional regulatory circuit in response to iron disturbance in iron-rich bacteria but also provides insights into the molecular mechanism through which phosphorylation-independent TCS response regulator functions.

## RESULTS

### Growth defect and lowered cyt *c* content of Δ*ssoR* are not attributed to impaired siderophore synthesis

**Fig 1:**
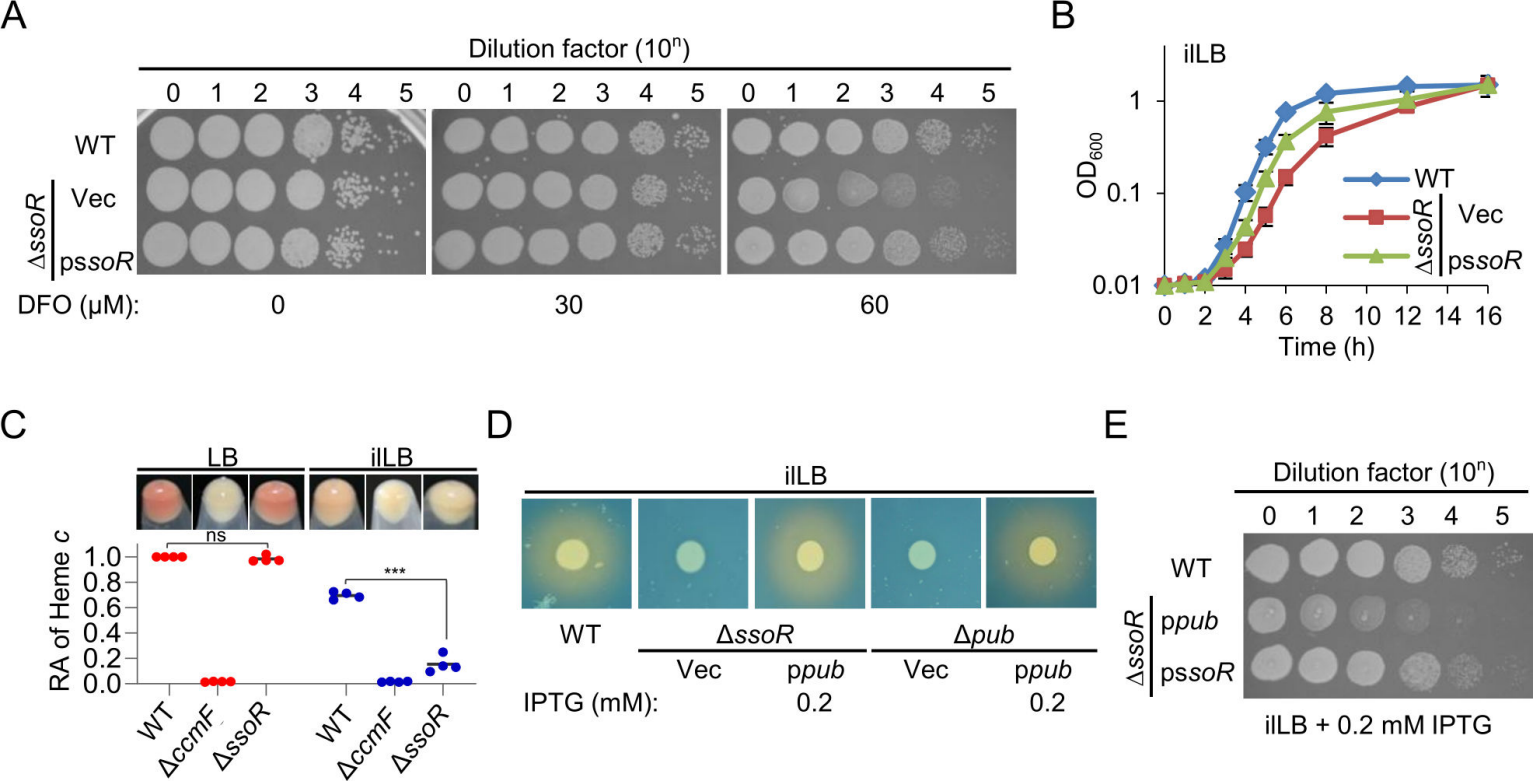
Physiological impacts of the *ssoR* mutation in *S. oneidensis*. (**A**) Plating efficiency of the *ssoR* mutant (∆*ssoR*) on LB plates supplemented with DFO at varying concentrations. Cultures prepared to contain approximately 10^8^ CFU/mL were regarded as the undiluted cultures (dilution factor, *n* = 0), which were subjected to 10-fold series dilution. Five microliters of each dilution was dropped on agar plates. All strains under test were cultured to mid-log phase (OD_600_≈ 0.4), and the results were photographed 18 h later. Vec, empty vector; p*ssoR*, expressing a copy of *ssoR* on a plasmid *in trans* for complementation. The gene was under the control of IPTG-inducible promoter P*tac* and the result shown was with 0.2 mM IPTG. (**B**) Growth of ∆*ssoR* in ilLB (LB supplemented with 60 µM DFO). (**C**) Colors of cell pellets and cytochrome *c* levels in the strains under test grown to the early stationary phase. The heme *c* levels of the WT and cytochrome *c* deficient strain ∆*ccmF* were set to 1 and 0, respectively. (**D**) Siderophore production assessed by CAS agar assay. Ten microliters of cultures (OD_600_ of ∼0.6) of the strains under test was dropped on LB agar plates. When comparable growth was reached, the CAS agar assay was performed and photographed after 3 h. (**E**) Growth of ∆*ssoR*-expressing operon *pub* under iron-depleted conditions. In all panels, experiments were independently performed at least four times. In panels A, D, and E, representative data were presented. In panel B, the data of four replicates were presented as the mean ± SD. In panel D, all of the data were presented. Statistical analysis was performed between indicated samples: ns, not significant; *, *P* < 0.05; **, *P* < 0.01; ***, *P* < 0.001.

To assess the impacts of SsoR on the iron physiology of *S. oneidensis*, we compared the growth of the wild-type (WT) and *ssoR* in-frame deletion strain (Δ*ssoR*) under normal and iron-limited conditions. Deferoxamine (DFO), a membrane impermeable iron chelator to *S. oneidensis* cells, was employed to create iron-limited conditions (21). While growth of the WT and Δ*ssoR* in lysogeny broth (LB) (normal condition) was found to be comparable, the mutant displayed a growth defect on LB agar plates supplemented with 60 µM DFO, named as iron-limited LB (ilLB; Fig. 1A). This defect could be attributed to reduced growth rate because, in liquid ilLB, the cell densities of the WT and Δ*ssoR* cultures eventually reached comparable levels (Fig. 1B). When a copy of *ssoR* was expressed *in trans*, the mutant restored growth almost undistinguishable from that of the WT, validating that SsoR underpins the observed phenotype (Fig. 1A and B).

We also noticed that iron limitation lightened the color of both the WT and Δ*ssoR* strains (cell pellets and colonies) apparently, and the effect was clearly much stronger on the mutant (Fig. 1C). Because of particularly abundant cyt *c* proteins that use iron-containing heme as a cofactor, the *S. oneidensis* WT is reddish-brown (Fig. 1C) (22). By quantifying heme *c*, we confirmed that Δ*ssoR* had a lowered cyt *c* content compared to the WT when grown in ilLB, and the difference became insignificant between the two strains grown in LB (Fig. 1C).

Based on both phenotypes, we reasoned that the *ssoR* mutant suffers from more severe iron starvation than the WT under iron-limited conditions. A convenient explanation for this proposal is the impaired siderophore-mediated iron uptake in the mutant because SsoR is a transcription activator for expression of the siderophore synthesis operon (15). To test this, the *pubABC* operon was forcibly expressed in ∆*ssoR* driven by an isopropyl β-D-1-thiogalactopyranoside (IPTG)-inducible promoter to varying levels. The expression was successful because ∆*ssoR* recovered the ability to synthesize siderophores, the same as a strain lacking the *pubABC* operon (∆*pub*; Fig. 1D; Fig. S1A). However, the growth defect of ∆*ssoR* that produced siderophores to varying levels under iron-deficient conditions was not significantly alleviated (Fig. 1E; Fig. S1B). Consistently, the manipulated expression of *pubABC* also failed to elevate cyt c content (Fig. S1C). These results rule out the possibility that the phenotypes of ∆*ssoR* under iron-limited conditions are due to impaired siderophore synthesis.

### SsoR is implicated in regulating genes involved in iron and heme homeostasis

**Fig 2:**
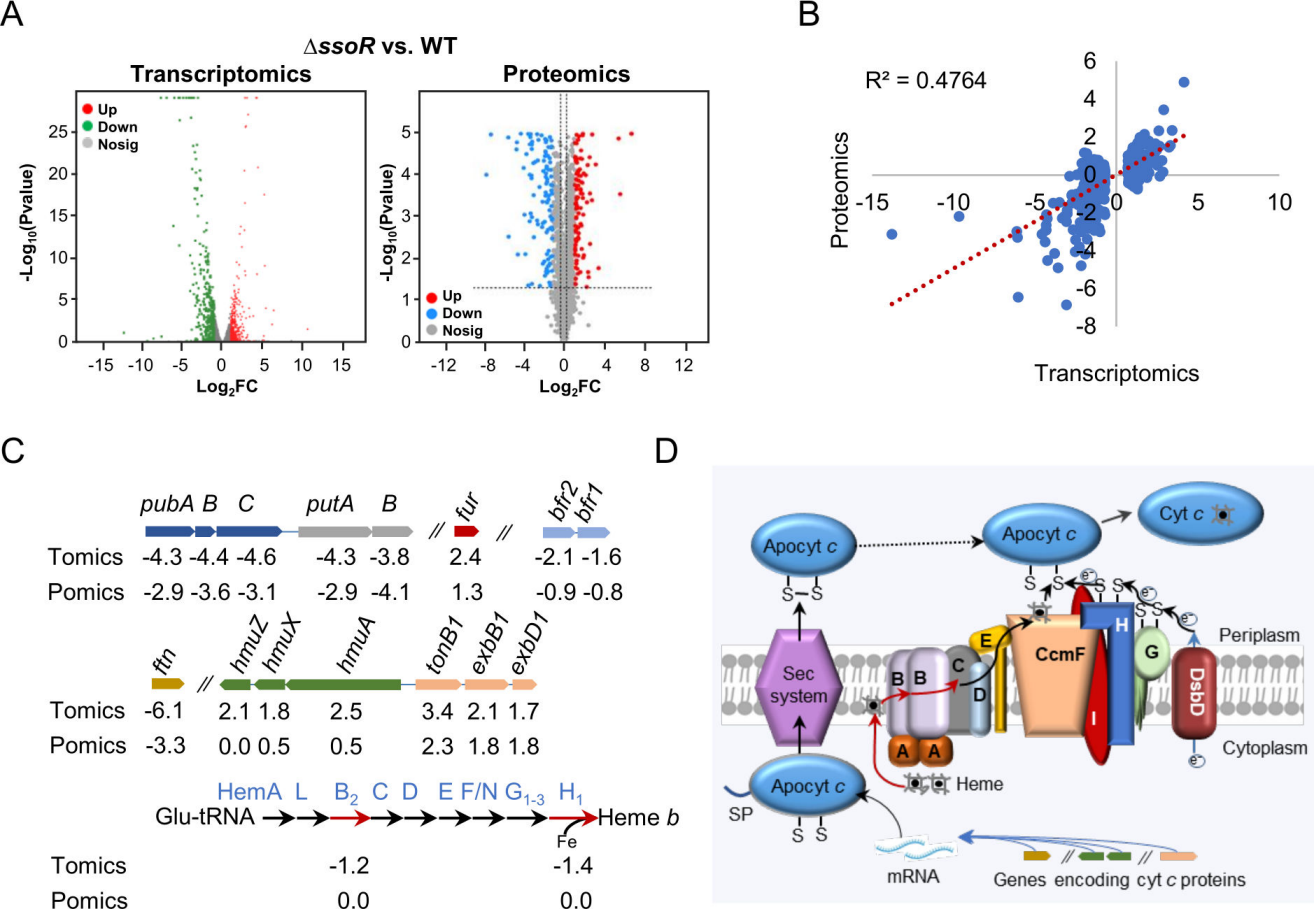
Co-omics analyses of the WT and ∆*ssoR* grown in ilLB. (**A**) Distribution of genes and proteins according to the expression difference between the WT and ∆*ssoR*. FC, fold change. (**B**) Correlation of the transcriptomics and proteomics data that passed the significant test by Pearson analysis. (**C**) Gene/proteins discussed and further investigated in this study. Tomics, transcriptomics; Pomics, proteomics. Genes in the same color are from a single operon; //, not linked on the chromosome. Genes/proteins in heme biosynthesis pathways are not dispersed on the chromosome. Arrows in red, transcriptomics data pass the significant test. (**D**) Schematic overview of cyt *c* maturation. After transcription and translation, cyt *c* genes produce apocyt *c* proteins, which enter the periplasm via the Sec system. These apocyt *c* proteins have to go through oxidation first, and then reduction by using electrons mediated CcmGH(I) and DsbD, which gets electrons from the cytoplasm. Heme ligation to reduced apocyt *c* proteins is catalyzed by CcmF after receiving heme from CcmABCDE.

To explore the mechanism underlying the defects of the ∆*ssoR* strain under iron-limited conditions, we carried out transcriptomics and proteomics profiling. To this end, the cultures of the WT and ∆*ssoR* grown to the mid-exponential phase in LB were shifted to ilLB, and samples were collected 10 and 30 min later for transcriptomic and proteomic analyses, respectively. In total, 427 genes in the transcriptomics data passed an ANOVA statistical analysis (*P* < 0.05) with Benjamini and Hochberg false discovery rate multiple testing correction between the WT and ∆*ssoR* strains, representing approximately 9.1% of the total ORFs (Table S1). In proteomics, to increase confidence of detection and quantitation, the proteins detected with more than two unique peptides were used for subsequent analyses. The transcriptomics and proteomics data display a similar pattern (Fig. 2A) and a Pearson correlation coefficient of 0.47 based on the genes/proteins that pass the significant test (Fig. 2B), suggesting that these two sets of data are correlated.

Among the genes that pass the significant test, nearly 70% (298 genes) were downregulated in the absence of SsoR, including those for many key metabolic enzymes, such as succinate dehydrogenase FrdCAB, formate dehydrogenases FdhABC and FdnEHIE, an expected scenario given that the ∆*ssoR* strain is impaired in growth under test conditions (Table S1). To stay focused, only genes/proteins that are implicated in iron physiology were discussed here. The *pubABC* operon, along with the *putAB* operon that encodes the specific TonB-dependent receptor and the reductase for uptake and reduction of siderophore-Fe^3+^, exhibited drastically reduced expression in ∆*ssoR* (Fig. 2C). This is in perfect agreement with the previous finding that the *pubABC* operon is one of the established members of the SsoR regulon (15), and a consistent reduction in expression of *putAB* can be readily explained by the coordinated regulation as these two operons are functionally linked (21, 23).

A large portion of the Fur regulon members was found to be heavily impacted by the SsoR loss (24) (Table 1). Out of the operons that carry a Fur-binding motif of high confidence (matrix weight ≥9) in front of the first gene of the operon, 19 were among the genes that passed the significant test, implying that Fur activity may be significantly altered. Indeed, both transcriptomics and proteomics data revealed that Fur is significantly more abundant in the *ssoR* mutant grown in ilLB (Fig. 2C). In *S. oneidensis*, Fur can work either as a repressor or activator, leading to the different expression patterns of these Fur-regulon members (24, 25). Despite this, it should be noted that major iron storage proteins Bfr1 and Bfr2 (24, 26), along with accessory iron storage protein Ftn, were heavily downregulated (Fig. 2C; Table S1).

**TABLE 1:** Expression difference of the Fur regulon in transcriptomics

Locus	Gene	Annotated function	Fold change (log2)^*a*^		Fur-binding motif weight^*b*^
			Tomics	Pomics	
SO_4819	SO_4819	Hypothetical protein			14.1
SO_2366	SO_2366	TCS response regulator			14.1
SO_4523	*irgA*	Iron-responsive TonB-dependent enterobactin receptor IrgA	2.81	0.74	13.2
SO_0744	*fbpA*	ABC-type Fe^3+^ uptake system substrate-binding component	1.31	0.46	12.6
SO_1482	SO_1482	TonB-dependent receptor			12.4
SO_3344	SO_3344	Predicted inner membrane protein	2.11		12.3
SO_4716	*ryhB*	Antisense RNA RyhB (SO_m007)	1.3		12.2
SO_1188	SO_1188	Inner membrane protein with PepSY TM helix	−3.87	−1.5	12
SO_4516	SO_4516	TonB-dependent siderophore receptor	−2.65	−1.22	11.8
SO_2064	SO_2064	Membrane-anchored YbdH domain protein			11.7
SO_3914	SO_3914	TonB-dependent siderophore receptor	2.86		11.4
SO_1111	*brf2*	Bacterioferritin subunit *2* Bfr2	−2.12	−0.9	11.4
SO_4685	SO_4685	Outer membrane protein in capsule/extracellular polymeric substance biosynthesis locus			11.2
SO_1998	SO_1998	Protein of unknown function DUF3389			11.2
SO_1427	*dmsE*	Periplasmic decaheme cytochrome c DmsE	−1.91	−2.11	10.9
SO_4740	SO_4740	Predicted membrane protein of unknown function DUF2061	3.51		10.8
SO_1813	*comEA*	DNA competence protein ComEA			10.8
SO_3670	*tonB*	Heme uptake energy transducer component TonB	3.42	2.36	10.3
SO_3669	*hmuA*	TonB-dependent heme/hemoglobin receptor HmuA	2.52	0.5	10.3
SO_3565	*cpdB*	2',3'-cyclic-nucleotide 2'-phosphodiesterase/3`-nucleotidase			10.1
SO_1755	SO_1755	Phosphoglucomutase/phosphomannomutase family protein	−1.84	−1.58	10
SO_0798	SO_0798	TonB-dependent receptor	1.52	0.06	10
SO_1966	SO_1966	Protein of unknown function DUF124			9.8
SO_1782	*mtrD*	Decaheme cytochrome c component MtrD	−1.25		9.7
SO_2841	SO_2841	Hypothetical protein	2.44		9.6
SO_2426	*ssoR*	Two-component signal transduction system response regulator	−13.8	−3.14	9.6
SO_1580	SO_1580	TonB-dependent haem/hemoglobin receptor			9.5
SO_1380	SO_1380	Protein of unknown function DUF2913			9.5
SO_4422	SO_4422	TonB-dependent ferric achromobactin receptor	1.02		9.4
SO_3549	SO_3549	Hemerythrin			9.4
SO_2247	SO_2247	Putative lipoprotein			9.4
SO_1911	SO_1911	Oxidoreductase short chain dehydrogenase/reductase family			9.4
SO_0139	*ftn*	Ferritin Ftn	−6.06	−3.31	9.4
SO_4196	SO_4196	Predicted membrane protein			9.3
SO_0554	SO_0554	Periplasmic protein of unknown function DUF3016			9.2
SO_3406	SO_3406	Putative siderophore transporter component 1	2.55	6.02	9

^*a*^ Values for genes that passed the significance test.

^*b*^ From reference 24.

We also paid particular attention to the genes encoding cyt *c* proteins because ∆*ssoR* has a reduced cyt *c* content when grown in ilLB. *S. oneidensis* has a repertoire of 42 cyt *c* proteins (27), and nearly half of its coding genes (~48%) were downregulated, such as *scyA*, *sorB*, *sorC*, *napB*, *dmsE*, *omcA*, *mtrD*, and *ccpA*, to name a few (Table S1). This suggests that expression of a large portion of cyt *c* genes is compromised. The cyt *c* content is also subjected to post-translational regulation, which refers to the heme attachment carried out by a dedicated protein system composed of 8–9 subunits (called cyt *c* maturation or biosynthesis system) in diverse gram-negative bacteria and archaea (28) (Fig. 2D). In gram-negative bacteria, the maturation occurs in the periplasm, requiring transmembrane transport of both the polypeptide chain (apocyt *c*) and heme, complicated oxidation and reduction of apocyt *c*, and efficient heme ligation, which have to be promptly carried out as apocyt *c* proteins are degraded rapidly (7, 29, 30). However, all components of the cyt *c* maturation system appeared to be unaffected by the SsoR loss (Table S1), suggesting that the lowered cyt *c* content is likely due to defects in other pathways, such as heme supply/traffic and compromised expression of apo-cyt *c* polypeptides (31, 32).

As heme is a key determining factor for the cyt *c* content and can be used as an iron source during iron shortage, we turned our attention to the genes involved in heme homeostasis (24). In transcriptomics, all of the genes in the heme uptake and utilization pathway, *hmuA* (TonB-dependent heme receptor), *hmuX* (heme binding protein), and *hmuZ* (heme oxygenase), as well as those for TonB-ExbB-ExbD that provide energy for heme uptake, were found to be upregulated (Fig. 2C). In addition, two out of nine genes (CcmB and CcmH) in the heme biosynthesis pathway were negatively affected. These data suggest the possibility that the Δ*ssoR* cells decrease heme production and increase heme degradation when grown in ilLB. Intriguingly, the products of these *hmu* and *ccm* genes were not found to be significantly different in proteomics (Fig. 2C), implying that expression of these genes may also be subjected to post-transcriptional regulation.

### Defects of ∆*ssoR* under iron-limited conditions are in part due to lowered heme levels

**Fig 3:**
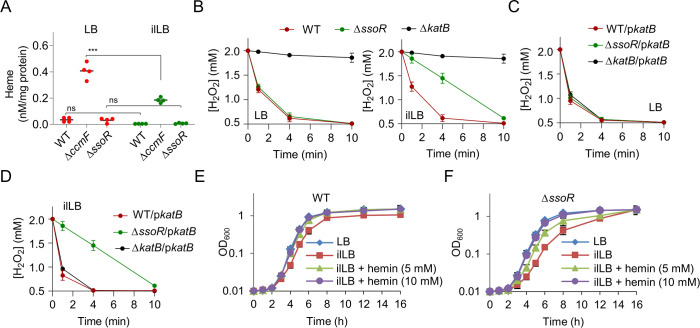
Heme is a crucial factor accounting for the phenotype of ∆*ssoR* grown in ilLB. (**A**) Intracellular heme concentrations of the relevant strains. Cells grown to the mid‐exponential phase were pelleted by centrifugation, and heme content was determined in cell lysates. Heme content was normalized to the protein concentration. (**B–D**) Catalase activity assay. H_2_O_2_ was added to mid‐exponential phase cultures to 0.3 mM, and concentrations of the remaining H_2_O_2_ after 1, 4, and 10 min were measured. (**E and F**) Growth of the WT and ∆*ssoR* strains with hemin addition. In all panels, experiments were independently performed at least four times. In panel A, statistical analysis was performed between indicated samples: ns, not significant; *, *P* < 0.05; **, *P* < 0.01; ***, *P* < 0.001. In panels B, C, and D, the data of four replicates were presented as the mean ± SD.

The omics data suggest the possibility that *S. oneidensis* Δ*ssoR* cells grown under iron-limited conditions may suffer from a heme shortage. Coincidentally, a similar phenotype of the impaired growth and lowered cyt *c* content was observed from the *S. oneidensis* strains that efflux heme (29). To directly test this, we attempted to measure intracellular heme concentrations in the WT and Δ*ssoR* strains grown under normal or iron-limited conditions. By using the QuantiChrom heme assay kit, we found that intracellular heme contents in these two strains grown under both conditions could not be confidently detected (Fig. 3A). In contrast, a cyt *c* deficient strain, ∆*ccmF*, which serves as the positive control because of its elevated heme levels (25), showed a significant reduction in heme levels when cultivated in ilLB, implying that the iron shortage generally leads to lowered heme concentrations.

To provide more confirmative results, we assessed intracellular heme concentrations by monitoring the activity of catalase KatB, a heme-dependent enzyme dictating H_2_O_2_ decomposition in *S. oneidensis* (33, 34). As *katB* expression levels of the WT and Δ*ssoR* grown in ilLB revealed in both transcriptomics and proteomics are not significantly different from each other (Table S1), the activity of the catalase would be correlated to the concentration of heme. The lysates of both the strains grown in LB decomposed H_2_O_2_ rapidly and in a comparable manner, in contrast to that of the negative control Δ*katB* (Fig. 3B). In contrast, when grown in ilLB, Δ*ssoR* was substantially compromised in scavenging H_2_O_2_ and also displayed substantially increased sensitivity to H_2_O_2_, compared to the WT (Fig. 3B; Fig. S2A). Moreover, we examined the effects of overexpressed *katB* on H_2_O_2_ decomposition rates of the WT and Δ*ssoR* strains grown under normal and iron-limited conditions. KatB in overabundance elevated the H_2_O_2_-decomposing rate of the WT grown in LB, as reported before (33, 34) (Fig. 3C). However, this effect was hardly observed from Δ*ssoR* grown under iron-limited conditions (Fig. 3D). Similar results were obtained from the assessment of H_2_O_2_ resistance of the WT and Δ*ssoR* overexpressing *katB* (Fig. S2A). These data strongly support that in the absence of SsoR, *S. oneidensis* cells suffer from heme shortage when cultivated under iron-limited conditions.

To provide direct evidence, we monitored the growth of Δ*ssoR* in ilLB with the addition of exogenous heme (hemin). While the addition of hemin did not show any detectable impacts on the growth of both WT and Δ*ssoR* under normal conditions (Fig. S2B), it could substantially improve the growth of both strains in ilLB (Fig. 3E and F). Hemin supplemented at 5 and 10 mM was sufficient to fully eliminate the growth difference of the WT and Δ*ssoR* caused by iron starvation, respectively. In addition, the hemin supplementation also restored the cyt *c* content of these strains to levels observed in their untreated counterparts (Fig. S2C). Taken together, these results conclude that the SsoR loss results in heme shortage when cells are grown in iron-limited conditions.

### Synthesis and degradation of heme are not the key pathways affected by the SsoR loss

**Fig 4:**
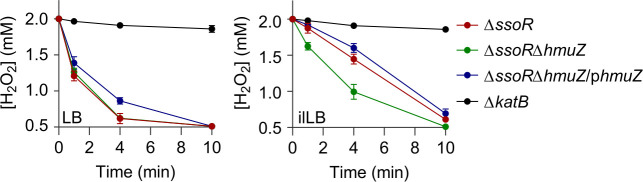
Heme degradation may have a role in heme shortage of ∆*ssoR*. Catalase activity assay was carried out as described for Fig. 3B through D. The experiment was independently performed at least four times, and the data of four replicates were presented as the mean ± SD.

Although there is a discrepancy between transcription and translation of the genes in heme synthesis and degradation, we attempted to determine if these processes are responsible for the heme shortage in Δ*ssoR* when grown in iron-limited conditions. To this end, the *hmuZ* gene, which encodes a heme oxygenase that dictates heme degradation in *S. oneidensis* (25), was removed from Δ*ssoR*, and the resulting double mutant was compared to Δ*ssoR* in terms of growth and H_2_O_2_ decomposition. In LB, the double mutant was not different from Δ*ssoR* on both accounts (Fig. 4; Fig. S3A). However, when grown in ilLB, the growth difference between these two mutants was insignificant (Fig. S3A). The double mutant decomposed H_2_O_2_ faster than Δ*ssoR*, suggesting that the heme degradation is implicated in the impacts of SsoR under an iron-limited condition (Fig. 4). This notion gained support from *hmuZ* overexpression, which slowed H_2_O_2_ decomposition and increased sensitivity to H_2_O_2_ (Fig. 4, Fig. S3B).

In the case of heme production, we assessed the effect of overexpression of *hemB*, *hemH*, or both together, two *hem* genes that were found to be downregulated in ∆*ssoR* according to the transcriptomics data. However, none of these attempts made a difference with respect to H_2_O_2_ susceptibility (Fig. S3B). We then overexpressed *hemA* as HemA (glutamyl-tRNA reductase) catalyzes the first dedicated, rate-limiting step in heme synthesis (35). Overexpression was successful because the heme content in the WT, ∆*ccmF*, and ∆*ssoR* strains grown in LB increased significantly as reported before (25) (Fig. S3C). However, the *hemA* expression did not cause a detectable change in H_2_O_2_ susceptibility of ∆*ssoR* when grown in ilLB (Fig. S3B). These data rule out the possibility that a heme biosynthesis pathway is a crucial factor associated with the physiological impact of SsoR under iron-limited conditions.

### Elevated Fur abundance is responsible for the defects of ∆*ssoR* under iron-limited conditions

**Fig 5:**
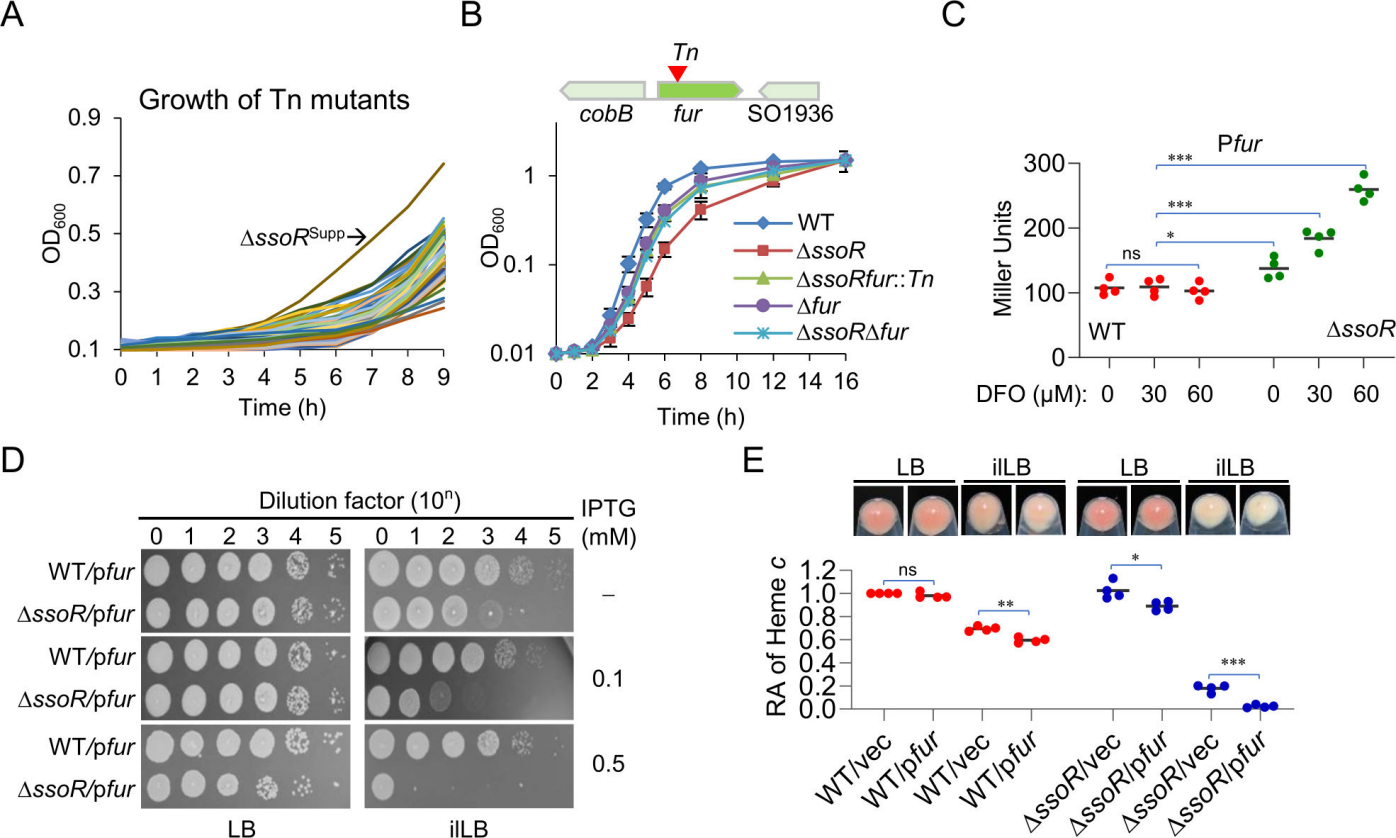
Elevated Fur abundance is responsible for the defects of ∆*ssoR* under iron-limited conditions. (**A**) Screening for suppressors of ∆*ssoR*. A transposon-insertion library of ∆*ssoR* was constructed and spread on 100 ilLB agar plates. Screening for suppressors (∆*ssoR*^Supp^) was described in the text. (**B**) A suppressor results from *fur* disruption. Genomic context of the *fur* locus in *S. oneidensis* with the transposon insertion sites marked. Growth of relevant strains in ilLB. (**C**) Expression of *fur* in indicated strains under different iron conditions by *fur*-LacZ reporter. Cells of the mid-exponential phase (OD_600_≈ 0.4) were collected for β‐galactosidase activity assay. (**D**) Effects of *fur* expression on growth. Expression was driven by IPTG-inducible promoter P*tac* with IPTG. (**E**) The effect of *fur* expression on cytochrome *c* contents. In panel B, the data of four replicates were presented as the mean ± SD. In panels C and E, statistical analysis was performed between indicated samples: ns, not significant; *, *P* < 0.05; **, *P* < 0.01; ***, *P* < 0.001. In panel D, representative data were presented.

To further address how the SsoR loss affects under iron-limited conditions, we attempted to identify suppressors that could correct the growth defect of ∆*ssoR*. A transposon-carrying vector was introduced into the ∆*ssoR* strain, generating a random library of ~20,000 mutants, from which we screened for those having improved growth as reflected by larger colonies on ilLB plates (100 plates). However, this strategy was found to be infeasible because the colonies of the library formed on ilLB plates in fact vary greatly in size. Instead, we pooled all colonies from each plate and cultivated the pools in ilLB. By comparing to ∆*ssoR*, we observed seven pools that showed improved growth. Then all isolates of these pools were grown in ilLB individually, and those that grew apparently faster than others (Fig. 5A) were subjected to validation with droplet assays (Fig. S4A).

In total, six isolates were obtained as ∆*ssoR* suppressor strains, in two of which transposon insertion sites were mapped to the *fur* gene (Fig. 5B). Two of the remaining suppressors had transposons that mapped within SO_0346 and SO_3743, which encode a GntR family transcriptional regulator and a TetR family transcriptional regulator, respectively; both of them remain functionally unknown. The sites of transposon insertion within the last two suppressors could not be determined, presumably due to multiple mutations. Within the first two suppressor strains (∆*ssoRfur*::Tn*)*, the coding region of the *fur* gene was interrupted by the transposon, rendering Fur unlikely to retain activity. To validate this, in terms of growth, we compared ∆*ssoRfur*::Tn and the ∆*fur*∆*ssoR* mutant strain that was constructed and verified previously (15). As expected, these two strains showed comparable growth under iron-limited conditions (Fig. 5B), supporting that Fur is required for the growth defect of ∆*ssoR* under iron-limited conditions.

Both transcriptomics and proteomics data revealed that the *fur* gene is upregulated in the absence of SsoR (Fig. 2A), which gained further support from results of the *fur* promoter activity assay with an integrative LacZ reporter (Fig. 5C). Clearly, expression of the *fur* gene in the ∆*ssoR* strain but not in the WT increased with DFO concentrations. We then examined whether enhanced Fur production is sufficient to cause the growth defect under iron-limited conditions. To this end, the *fur* gene was expressed to varying levels in both the WT and ∆*ssoR* strains. Results demonstrated that the impact of overexpressed Fur on growth of both the strains was negligible under normal conditions (Fig. 5D). Under iron-limited conditions, while the susceptibility of ∆*ssoR* to Fur increased with IPTG concentrations, the WT was found to be recalcitrant to the changes in Fur levels (Fig. 5D). Consistently, we noticed that Fur in excess reduced the cyt *c* content of the WT and ∆*ssoR* strains when grown in ilLB (Fig. 5E). This effect was even significant in ∆*ssoR* cells grown in LB (Fig. 5E).

Elevated *fur* expression in the *ssoR* mutant suggests the possibility that SsoR may repress transcription of the *fur* gene directly, in contrast to its role as an activator for the *pub* operon (15). To test this, the *in vivo* interaction between SsoR and the *fur* promoter region sequence was assayed in *E. coli* with a bacterial one-hybrid (B1H) system (36). Vectors containing “bait” (DNA) and “target” (DNA-binding regulator) are co-transformed into BacterioMatch II Validation Reporter Competent Cells, of which those having positive DNA-protein interactions are able to grow on 3-amino-1,2,4-triazole (3-AT). While strong interaction was observed in the positive control (pTRG-P*pub*/PBXcmT-*ssoR*), no interacting signal was observed between the *fur* promoter component and SsoR (Fig. S4B), eliminating the possibility that SsoR regulates *fur* transcription directly. These data, all together, suggest that the detrimental effects of excess Fur in *S. oneidensis* are evident in the depletion of SsoR and become substantially more severe upon iron starvation.

### Apo-Fur of *S. oneidensis* lacks significant regulatory activity

**Fig 6:**
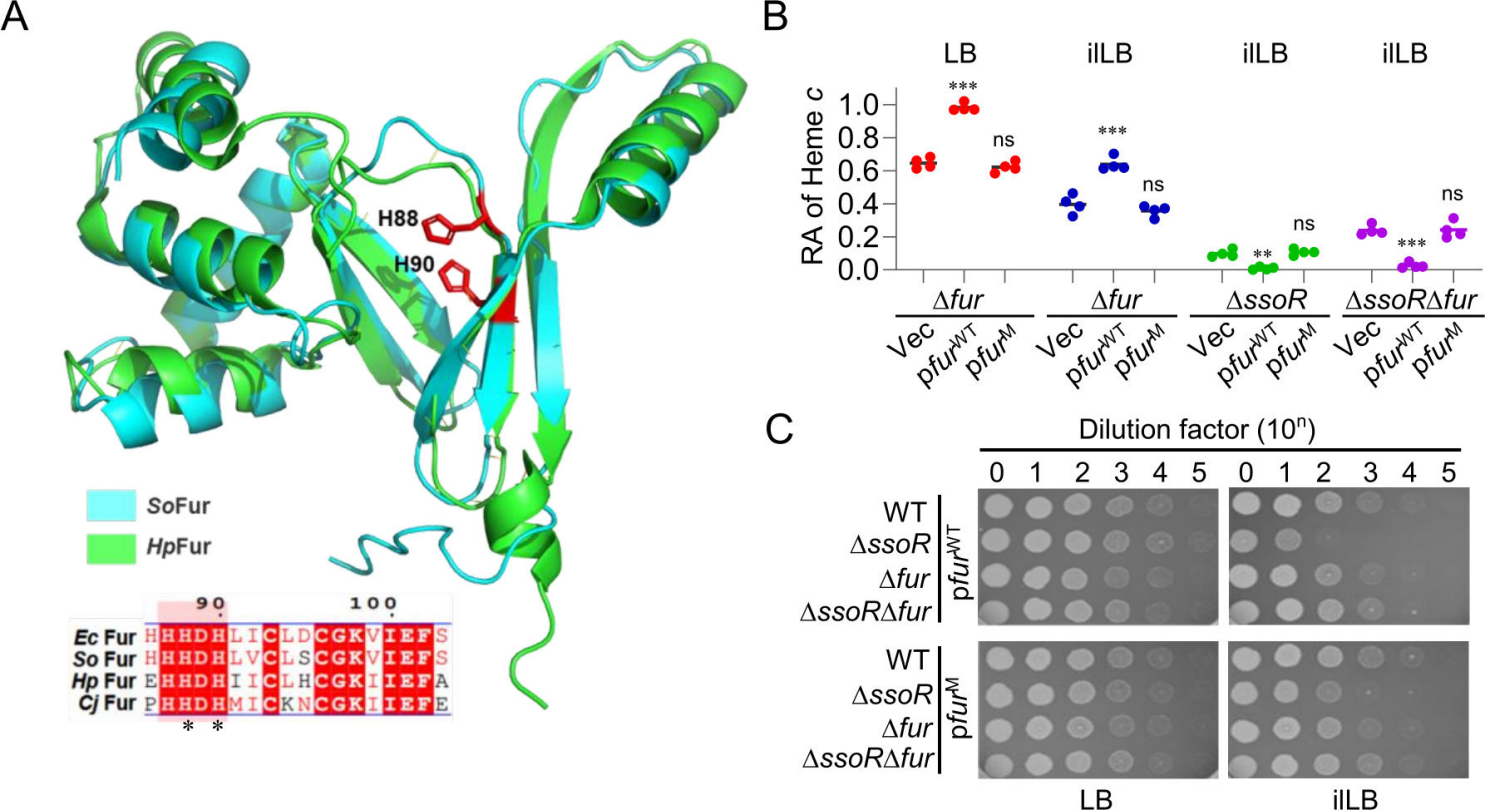
Apo-Fur of *S. oneidensis* lacks significant regulatory function. (**A**) The monomer structural comparison of Fur proteins from *S. oneidensis* (*So*Fur) and *H. pylori* (*Hp*Fur, PDB: 2XIG), which could function in the form of apo-Fur*. So*Fur is predicted by AlphaFold3. Two histidine residues essential for iron binding are shown. Inset, the highly conserved region of the Fur proteins from *E. coli* (*Ec*Fur), *So*Fur, *Hp*Fur, and *C. jejuni* (*Cj*Fur), taken from Fig. S5B. The residue numbering is based on *Ec*Fur and *So*Fur, which are identical. The residues subjected to mutational analysis are marked with an asterisk (*). (**B**) Impacts of Fur loss and Fur^M^ on cyt *c* levels in the strains under test grown to the early stationary phase. M represents the H88L/H90L double mutation. (**C**) Impacts of Fur loss and Fur^M^ on the growth of the strains under test. In panels B and C, the genes of interest were under the control of IPTG-inducible promoter P*tac*, and the result shown was with 0.2 mM IPTG. All experiments were independently performed at least four times. In panel B, all of the data were presented. Statistical analysis was performed between cells carrying vec and expressing *fur*^WT^ or *fur*^M^: ns, not significant; *, *P* < 0.05; **, *P* < 0.01; ***, *P* < 0.001. In panel C, representative data were presented.

Although Fur is commonly regarded as a Fe-dependent global regulatory factor, in some bacteria, such as *Helicobacter pylori* and *Campylobacter jejuni*, Fur can exert regulatory function in the apo-Fur form (9–11). To test if excess Fur in its apo-Fur form is responsible for increased sensitivity of ∆*ssoR* to iron deficiency, we performed *in silico* analysis of the canonical Fur proteins that are iron-dependent and those functioning as apo-Fur. Sequence similarity network of Fur homologs revealed that Fur proteins are rather diverse and assemble a large number of genus-specific clusters, including *Helicobacter*, *Shewanella*, *Campylobacter*, and *Vibrio*, to name a few (Fig. S5A). In addition, Fur proteins share relatively low sequence similarity; for example, sequence identity of Fur of *S. oneidensis* and *H. pylori* Fur (*Hp*Fur) is 27.8% (Fig. S5B). Despite this, Fur proteins are not only highly similar in secondary structure arrangement and 3-D structure but also share a conserved HHDH motif for binding iron (Fig. 6A; Fig. S5B), suggesting that they all employ a similar mechanism for interacting with iron (37).

It has been established that the two histidine residues, H88 and H90 in the case of both *E. coli* and *S. oneidensis*, are essential for iron binding (4, 38) (Fig. 6A). A mutant Fur^H88L/H90L^ was then constructed and expressed in relevant strains. In contrast to Fur^WT^, expression of Fur^H88L/H90L^ failed to complement the phenotype of the lowered cyt *c* content resulting from the Fur loss in the WT background (24) (Fig. 6B). Consistent with the suppression of growth defect of ∆*ssoR* grown in ilLB, the additional Fur loss elevated the cyt *c* content significantly, and this effect could only be neutralized by expression of Fur^WT^ (Fig. 6B). Additionally, excess Fur^H88L/H90L^ could exacerbate the defects neither in the cyt *c* content nor in growth of ∆*ssoR* (Fig. 6B and C). All of these data conclude that Fur^H88L/H90L^ could not act as a functional replacement of Fur^WT^, suggesting that apo-Fur in *S. oneidensis* is unlikely to play a significant role in physiology.

### Disrupted iron homeostasis is in part accountable for the defects of the *ssoR* mutant under iron-limited conditions

**Fig 7:**
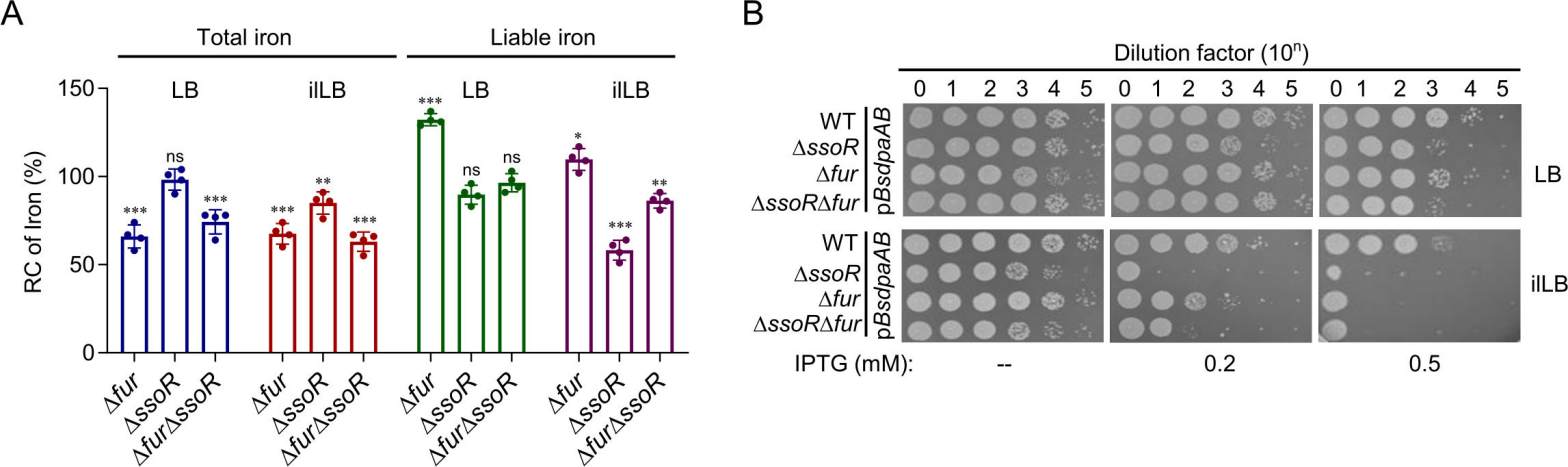
The SsoR loss results in lowered total and labile iron levels. (**A**) Effects of the SsoR and Fur on cellular iron levels. Cultures of indicated strains entering the stationary phase were collected, and their total iron and labile iron contents were determined. The averaged levels of the WT were set to 100%. (**B**) Impacts of endogenous iron chelator on the growth of the strains under test. p*BsdpaAB*, *Bacillus subtilis dpaAB* gene under the control of Ptac. In all panels, experiments were independently performed at least four times. In panel A, all of the data were presented. Statistical analysis was performed between indicated mutants and the WT: ns, not significant; *, *P* < 0.05; **, *P* < 0.01; ***, *P* < 0.001. In panel B, representative data were presented.

The Fur loss in *S. oneidensis* results in disruption of iron homeostasis, with lowered total iron content but elevated labile iron concentrations (24, 25). Iron homeostasis is likely to be disrupted with the SsoR depletion, given that Fur is in excess and major iron storage bacterioferritin Bfr is downregulated heavily in ∆*ssoR* (Fig. 2C). To directly assess this, ferrozine-based colorimetric assay and electron paramagnetic resonance spectroscopy were employed for quantification of total cellular iron and labile iron, respectively. Under normal growth conditions, the impacts of the SsoR loss on both total and labile iron levels were negligible (Fig. 7A). In contrast, when grown in iron-limited conditions, the Δ*ssoR* strain contained modestly, but significantly, lowered levels of total iron and substantially reduced concentrations of labile iron (Fig. 5A). Additionally, labile iron levels in Δ*ssoR* were assayed with streptonigrin (SNG), a redox cycling antibiotic whose antibacterial activity correlates with the levels of intracellular free iron (39). The results show that the Δ*ssoR* strain exhibited higher resistance to SNG than the WT (Fig. S6A), supporting that the mutant has a low labile iron content.

Furthermore, we produced endogenously dipicolinate in *S. oneidensis*, an iron chelator that is unable to cross cell membranes and therefore could substantially reduce labile iron levels in bacteria (40). Dipicolinate can be synthesized by *Bacillus subtilis* dipicolinate synthetase (encoded by *BsdpaAB*) through a one-step reaction from dihydrodipicolinate, an intermediate in the pathway of diaminopimelate and lysine biosynthesis (Fig. S6B). We reasoned that the impacts of *BsdpaAB* expression on growth of *S. oneidensis* strains whose labile iron concentrations are significantly different could be differentiated. When *BsdpaAB* was expressed with 0.5 mM IPTG, even growth of the WT under normal conditions was noticeably impaired, and this impact was more evident for strains ∆*ssoR*, ∆*fur*, and ∆*ssoR*∆*fur* (Fig. 7B). Under iron-limited conditions, ∆*ssoR* became hypersensitive to *BsdpaAB* expression compared to other strains under test, seemingly correlated to their labile iron levels. Intriguingly, although the *fur* mutant has a higher labile iron level than the WT (Fig. 7A), it was apparent that the WT was more resistant to *Bs*dpaAB production, likely due to the strain being capable of regulating iron homeostasis. These data, all together, conclude that the reduction in labile iron, likely together with the reduction in total iron, is largely accountable for the physiological impacts of the SsoR loss under iron-limited conditions.

## DISCUSSION

**Fig 8:**
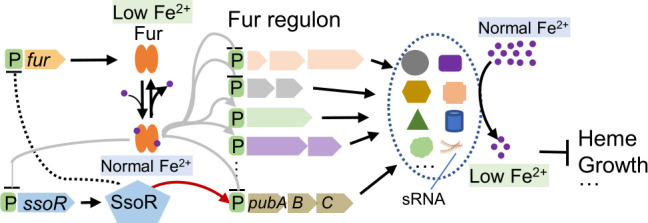
A model for the derepressing-inhibiting oscillation regulation by Fur and SsoR. Iron starvation (low Fe^2+^), which reduces heme levels and growth rate, results in altered expression (solid lines in gray, either repression or activation) of the Fur regulon. When Fur proteins are present at proper levels, this regulation would increase iron uptake and reduce iron disposal, thereby maintaining iron homeostasis. However, when Fur proteins are in excess, the ratio of Fur to Fe^2+^ is likely increased, leading to distorted expression of the Fur regulon, which in this case reduces intracellular iron levels. Iron starvation may further aggravate the situation by inactivating more Fur proteins. SsoR, repressed by Fur directly, would be overproduced in the presence of excess Fur. As SsoR inhibits *fur* expression indirectly (dashed line in black), the more the SsoR proteins, the stronger the inhibition, which can eventually ensure that both Fur and SsoR oscillate back to normal levels. Red arrow line, activation.

Bacterial iron homeostasis is maintained through a multi-layered regulatory network involving transcription factors (Fur, DtxR), sRNAs (RyhB, PrrF), Fe-S cluster sensors (IscR), and multiple TCSs, which ensures optimal iron levels for metabolism while minimizing oxidative damage (5, 41). This is particularly critical for bacteria that have a markedly higher iron demand and sensitivity to iron depletion compared to others, such as *Shewanella*, which express a large repertoire of iron-containing proteins to support highly branched electron transport pathways for respiration of numerous diverse substrates (17, 19). In addition to Fur, a couple of TCSs of *S. oneidensis* have been implicated in the regulation of iron homeostasis, including BarA/UvrY and SsoR (15, 42, 43). While the regulation of BarA/UvrY as a canonical TCS occurs through transcriptional and post-translational mechanisms that respond to shifts in carbon metabolism or some secondary metabolite processes, SsoR represents a novel TCS response regulator that functions in a phosphorylation-independent manner (15). In this study, we present evidence suggesting that SsoR and Fur are highly intertwined to mediate iron homeostasis, as the loss of either leads to increased production of the other, affecting iron homeostasis (Fig. 8). This complexity likely represents an adaptive regulatory strategy evolved in bacteria, enabling them to function effectively without relying on a single limiting factor (1, 3).

Under iron-limited conditions, the SsoR loss results in increased susceptibility to iron shortage, leading to lowered growth rate and cyt *c* content. As the genes for the siderophore synthesis system depend on SsoR for transcription (15), it is reasonable to assume that the lack of siderophores would impair the *ssoR* mutant to uptake iron in the iron-limited environment and thus lead to the observed phenotypes. However, this is not the case, implying that the underpinning mechanism is beyond iron uptake. Indeed, the transcriptomics and proteomics analyses reveal that many genes encoding proteins involved in iron homeostasis, including iron storage, heme synthesis and degradation, cyts *c*, and iron-responsive regulation, are differentially expressed. In particular, a large portion of the Fur regulon members are among the genes that show most significantly altered expression (24).

*S. oneidensis* can grow only through respiration, and the respiration of all electron acceptors known to date, including oxygen and non-oxygen ones, is absolutely dependent on hemoproteins, rendering heme to be an essential molecule (22, 44). Decreased and increased expression of genes in heme synthesis and degradation, respectively, revealed by the omics data, resonates with our previous finding that the lowered intracellular heme levels would result in the impaired growth and lowered cyt *c* content (29). By assessing the activity of heme-dependent catalase KatB, which plays a predominant role in H₂O₂ decomposition in *S. oneidensis* (33, 45), we demonstrated that the *ssoR* mutation significantly lowers heme levels when the cells are grown under iron-limited conditions. This notion gains further support from the fact that the defect in growth and cyt *c* content can be corrected largely by the addition of exogenous heme (hemin). Interestingly, in the *ssoR* mutant, while heme degradation plays a modest role in lowering heme levels, the impact of heme synthesis is negligible. Similar scenarios have been observed before in strains lacking Fur or overexpressing Hfq (21, 23, 25, 46), suggesting that heme degradation is likely a common route for fine-tuning intracellular heme concentrations. It should be noted that there is a discrepancy between transcription and translation of genes involved in heme synthesis and degradation. This may not be surprising given that both Hfq and RyhB have been shown to have a role in regulating heme levels and abundance of hemoproteins, such as cyts *c* (46–48).

Although *fur* is upregulated in the *ssoR* mutant grown under iron-limited conditions, it is transposon mutagenesis screening for suppressors that solidifies the crucial contribution of Fur to the defect caused by the SsoR loss. Disruption of the *fur* gene effectively corrects the growth defect of the *ssoR* mutant under iron-limited conditions. More importantly, we provide evidence that further increased expression of the *fur* gene worsens the defects in growth and cyt *c* biosynthesis of the *ssoR* mutant under iron-limited conditions. However, the *fur* gene, unlike the *pub* operon (15), is not transcriptionally regulated by SsoR directly.

Given the repressing role of Fur for iron acquisition, the Fur loss commonly leads to iron overload in bacteria (3). Although a large portion of these studies does not distinguish between labile iron and iron bound to proteins, it is reasonable to assume that the free iron content increases, as revealed in *Enterococcus faecalis* (49). Given that in *S. oneidensis*, as well as in *E. coli*, the loss of Fur results in the lowered total and elevated labile iron contents (25, 50), one may imagine that Fur in excess caused by the SsoR loss would lead to reversion of this trend. However, it turns out that the *ssoR* mutant suffers from the lowered iron contents in terms of both the total and the labile. While this notion is consistent with the substantial reduction in expression of iron storage proteins Bfr and Ftn (26), the direct support comes from the manipulated production of membrane-impermeable iron chelator, which further lowers intracellular iron levels and thereby worsens growth.

In *S. oneidensis*, the impacts of Fur on biological processes have expanded beyond those related to iron, including the induction of λSo prophage and the production of outer-membrane porins (51, 52). Despite this, *S. oneidensis* Fur lacks regulatory function in the apo-Fur state. This may not be surprising because, to date, apo-Furs that are able to directly regulate gene expression by binding the apo-Fur boxes have only been confirmed in very few bacterial species (11)

Perhaps one of the most unexpected fruits from this work is that Fur and SsoR constitute a transcriptional derepressing-inhibiting system to regulate iron homeostasis (53) (Fig. 8). Our previous research has revealed that SsoR is not phosphorylation-dependent but dose-dependent to regulate the transcription of its regulon (15). Instead of a cognate histidine kinase for SsoR, Fur senses reduction in intracellular iron levels and derepresses *ssoR* transcription (15). As SsoR is constitutively active, to avoid damages caused by the protein in excess, SsoR, in addition to repressing its own transcription (15), inhibits *fur* expression, albeit indirectly, to prevent Fur from overexpression (Fig. 8). One can imagine that Fur in excess could lead to an increase in the ratio of Fur to iron molecules and may trigger a signal of iron shortage, resulting in more SsoR proteins to be produced (Fig. 8). Clearly, the inhibitory role of SsoR in this Fur/SsoR derepressing-inhibiting system may eventually help both Fur and SsoR to oscillate back to normal levels. This may be particularly important for *Shewanella* species as their unusually high iron content not only supports their respiratory versatility but also jeopardizes their survival and proliferation (19).

## MATERIALS AND METHODS

### Bacterial strains, plasmids, and culture conditions

The strains and plasmids used in this study are listed in Table S2. The chemicals used were obtained from Sigma-Aldrich unless otherwise noted. For genetic manipulation, *E. coli* and *S. oneidensis* strains were grown under aerobic conditions in Lennox LB (Difco, Beijing, China) at 37°C and 30°C, respectively. Chemicals added to the growth medium as needed for the experiments are as following concentrations: 2,6-diaminopimelic acid (DAP), 0.3 mM; ampicillin sodium, 50 µg/mL; kanamycin sulfate, 50 µg/mL; gentamicin sulfate, 15 µg/mL; DFO, 30 µM; and IPTG, 0.1 M.

### In-frame mutant construction and complementation

In-frame deletion mutants were constructed using homologous recombination involving *att*-based fusion PCR and SacB-based counterselection (27). In brief, the vector for *fur* deletion, from *E. coli* WM3064 (DAP auxotrophic) (52), was transferred into the *S. oneidensis* strain via conjugation. Gentamicin-resistant colonies were selected, and integration of the vector into the chromosome was confirmed by PCR. Verified strains were then cultured in NaCl-free LB, diluted, and plated onto LB agar containing 10% sucrose. Colonies that were gentamicin-sensitive and sucrose-tolerant were screened by PCR for deletions of the target genes, and final verification was carried out by sequencing the region containing the intended mutations.

The genetic complementation of in-frame deletion mutants and manipulated gene expression was performed using pHGEN-P*tac*, in which the introduced gene was transcriptionally driven by IPTG-inducible promoter P*tac* (54). Sequencing-verified complementation vectors were first transformed into *E. coli* WM3064 and then were transferred into the relevant *S. oneidensis* strains via conjugation.

### Bacterial growth assessment

Both normal LB and ilLB, which contain 60 µM DFO, as well as defined media MS and ilMS (with 60 µM DFO), which is a 1,4-piperazinediethanesulfonic acid (PIPES)-buffered minimal salts medium containing 30 mM lactate as electron donor (55), were used in this study. While similar results were obtained from the complex and defined media, growth in ilMS was extremely slow, prompting us to use the complex media throughout this study. Growth of *S. oneidensis* strains in liquid media under aerobic conditions was measured at 600 nm (OD_600_). Fresh media were inoculated with overnight cultures by 200-fold dilution (OD_600_, ~0.01) and shaken at 200 rpm at 30°C. In parallel, droplet assays were adopted to assess growth on agar plates. Cultures grown in liquid media to the mid-exponential phase were adjusted to approximately 10^8^ CFUs/mL and followed by 10-fold serial dilutions. Ten microliters of each dilution was spotted onto plates, which were incubated at 30°C for 24 h before being read.

### Transcriptomics and proteomics profiling

The transcriptome and proteome were profiled to reveal diﬀerences in transcripts and proteins resulting from the *ssoR* mutation. For both omics experiments, the wild-type and Δ*ssoR* strains of three biological replicates grown to the mid-exponential phase in LB and ilLB were collected by centrifugation, stored in liquid nitrogen before use. For transcriptomic profiling, the cells were suspended in Trizol, and then total RNA was isolated following the protocol of the QIAGEN RNase Mini kit (Valencia, CA, USA) as described before (33). For proteomic profiling, the pellets were lysed in 4% sodium deoxycholate after washing in phosphate-buffered saline (PBS). Lysates were washed, reduced, alkylated, and trypsinized as described before (56). Transcriptome sequencing and tryptic peptides identification, as well as subsequent preliminary analyses, were performed by Shanghai Majorbio Bio-pharm Technology Co., Ltd.

### Transposon mutagenesis

Plasmid pHGT01 was used to construct transposon insertion mutants, containing a mariner transposon element that can randomly insert into the bacterial genome upon expression (57). The plasmid was transferred to the target strain of *S. oneidensis* via conjugation from *E. coli* WM3064. After 12 h, cells were washed off the conjugation plates with LB, properly diluted, and spread on 100 LB agar plates containing gentamicin. All colonies of each plate were inoculated into ilLB medium and shaken at 200 rpm at 30°C. From the plates that showed better growth, all colonies were then grown individually. Those that grew faster were then chosen for transposon insertion mapping as described before (32).

### Expression assay

A single-copy integrated *lacZ* reporter system was used to evaluate the activity of target promoters (58). Briefly, a fragment containing up to 500 bp upstream of the target gene coding sequence was amplified and cloned into the reporter vector pHGEI01. The resulting vector was transformed into *E. coli* WM3064, and after verification by sequencing, was then transferred by conjugation into relevant *S. oneidensis* strains, allowing integration of the reporter construct into the chromosome, after which the antibiotic marker was removed (59). The promoter activity was assayed from the target strains grown to the mid-exponential phase and was performed with a β-galactosidase activity assay kit from Solarbio (Beijing) according to the manufacturer’s instructions. Throughout this study, protein concentrations of the cell lysates were determined by the bicinchoninic acid assay (Pierce Chemical).

### Site-directed mutagenesis

Site-directed mutagenesis was performed as described before (45). Genes of interest within pHGEN-P*tac* were applied to site-directed mutagenesis with Quick-Change Kit (Agilent) according to the manufacturer’s guidelines. All substitutions were verified by DNA sequencing.

### Siderophore assays

Siderophore assays were performed essentially the same as described before (15). In brief, 10 µL of bacterial cultures grown to the mid-exponential phase was spotted onto LB or ilLB agar plates and incubated for 18 h. CAS detection reagent was applied to the plates with bacterial colonies, and the yellow halos were recorded after 30 minutes.

### Catalase activity assay

Catalase activity was assessed with the H_2_O_2_ consumption assay, essentially the same as described before (34). Briefly, mid-exponential-phase cells in liquid medium were collected, washed twice in 50 mM KH_2_PO_4_ buffer (pH 7.0), resuspended in the same buffer, and then disrupted by sonication. The cell extracts containing 40 ng/µL protein were added to 90 µL of KH_2_PO_4_ and 100 µL of 2 mM H_2_O_2_ in a 200 µL volume. Decomposition of H_2_O_2_ by the FOX-assay method was measured at 240 nm with absorbance readings taken at 15 s time intervals for a total time of 10 min in a Tecan M200 Pro microplate reader.

### Disc diffusion assay

The disc diffusion assay was performed as previously described (60). Briefly, 200 µL mid-exponential phase cultures were spread onto agar plates, and the plates were incubated at 30°C. A certain period later, depending on the growth rate of the strain under test, paper discs of 6 mm in diameter loaded with the chemical at the desired concentrations under test (H_2_O_2_, streptonigrin) were placed onto the bacterial lawn. The plates were incubated at 30°C for 20 h or longer, and the diameter of the zone of growth inhibition was measured to assess the sensitivity of the cells against the agent.

### Heme quantification assay

Cultures of *S. oneidensis* strains grown to the early stationary phase were aliquoted to contain similar numbers of cells and centrifuged. The resulting pellets were photographed and subsequently subjected to heme quantification. Total heme and heme c were quantified with QuantiChrom heme assay kit (BioAssay Systems), essentially the same as described before (29, 32). The standard curve was generated with heme solutions from 4 to 150 nM. For heme *c* measurement, the proteome of the culture was extracted and denatured by trichloroacetic acid (8%, final concentration) precipitation to release non-covalently attached heme, and the precipitated protein portion was assayed. The intracellular heme content was derived from the difference in concentrations of total heme and heme *c*. Cyt *c* deficient strain, Δ*ccmF*, was included as a negative control in each experiment, whose values were taken as the background. The heme *c* levels of the strains under test, after subtracting the background, were normalized against the average level of the WT, generating relative abundance.

### Quantification of intracellular total and labile iron species

The content of intracellular total and labile iron species was assessed by the established methods (55, 61). To measure total iron, cells grown to the stationary phase were pelleted, washed with PBS (pH 7.4), adjusted to similar densities (OD_600_, ~0.6), and sonicated. The cell lysates of 100 µL were mixed with 100 µL 10 mM HCl and 100 µL iron releasing reagent (a freshly mixed solution of equal volumes of 1.4M HCl and 4.5% [wt/vol] KMnO_4_) and treated at 60°C for 2 h. After cooling, the iron detection reagents (6.5 mM ferrozine, 6.5 mM neocuproine, 2.5 M ammonium acetate, and 1 M ascorbic acid in water) were added. The absorbance of samples was measured at 550 nm 30 min later. The standard curve was developed using FeCl_3_ up to 300 µM. To measure labile iron, aliquots of cells were mixed with 5 µM calcein-AM (acetomethoxy derivative of calcein) for 30 min at 37°C. At the same time, other aliquots were treated with the iron chelator dipyridyl for 10 min prior to treatment with calcein-AM for comparison. Cells were then washed with PBS to remove the extracellular calcein-AM and monitored for signal changes using a Tecan M200 pro microplate reader (excitation at 485 nm and emission at 535 nm).

### B1H assay

B1H system was used to investigate DNA-protein interaction *in vivo* in *E. coli* cells, essentially the same as described before (36, 62). Briefly, plasmid constructs were created by cloning the bait DNA (promoters under test) and target SsoR in the pBXcmT and pTRG vectors and verified by sequencing. The resultant plasmids were used to co-transform BacterioMatch II Validation Reporter Competent Cells on M9 salt agar plates containing 25 mg/mL chloramphenicol and 12.5 mg/mL tetracycline with or without 3-AT. The plates were incubated for 24 h and then moved to room temperature for an additional 16 h (the colonies indicating positive interaction usually appeared between 18 and 24 h). The positive interactions were confirmed by streaking colonies on plates containing both 3-AT and streptomycin (12.5 mg/mL).

### Bioinformatics and statistical analyses

The protein amino acid sequence alignment in this study was performed using the Clustal Omega online tool. The structure of the Fur of *S. oneidensis* was predicted with AlphaFold2 (63). The structural alignment between relevant Fur proteins was carried out using PyMOL software. In this study, experiments were performed with at least three independent replicates, and the data are presented as mean ± SD. Student’s *t*-test was used to analyze the differences between two groups of data.

## Data Availability

The data underlying this article are available in the article and its supplemental material. Other data generated during and/or analyzed during the current study are available upon request from the corresponding author. The raw RNA sequencing data have been deposited to the National Center for Biotechnology Information’s Sequence Read Archive database with the BioProject number PRJNA1255826, and the mass spectrometry proteomics data have been deposited to Integrated Proteome Resources (iProX) with project ID IPX0011865000 (PXD063471).

## SUPPLEMENTAL MATERIAL

Supplemental material aem.01230-25-s0001.pdf Figures S1 to S6; Tables S1 and S2.

ASM does not own the copyrights to Supplemental Material that may be linked to, or accessed through, an article. The authors have granted ASM a non-exclusive, world-wide license to publish the Supplemental Material files. Please contact the corresponding author directly for reuse.
