# Supplementary material for: Regulation of iron homeostasis by Fur and atypical response regulator SsoR via derepressor-inhibitor oscillation in Shewanella oneidensis
Source: Appl Environ Microbiol. 2025 Aug 27;91(9):e01230-25. doi: 10.1128/aem.01230-25 (PMC12442407; doi:10.1128/aem.01230-25)
Supplement: Supplemental material — Figures S1 to S6; Tables S1 and S2. [file aem.01230-25-s0001.pdf]

Supporting Information for

Regulation of iron homeostasis by Fur and atypical response regulator SsoR via derepressor-inhibitor oscillation in *Shewanella oneidensis*

Kaiyue Jie,[#] Xinyue Liu,[#] Jiyuan Hou, Peilu Xie, Jiaxin Tang, and Haichun Gao*

*Corresponding authors: Haichun Gao (haichung@zju.edu.cn)

This PDF file includes:

Supplementary Figures S1 to S6

Supplementary Tables S1 to S2

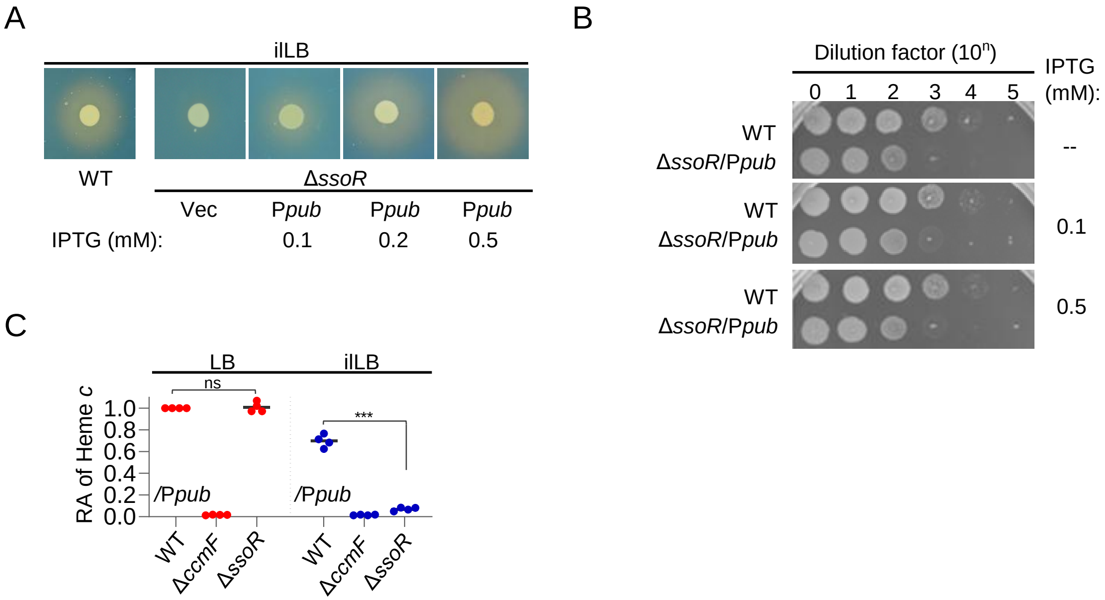

FIG S1. The effects of *pub* expression in Δ*ssoR*. (A) Siderophore production assessed by CAS agar assay. Ten microliter of cultures (OD_{600} of ~0.6) of the strains under test was dropped on LB agar plates. When comparable growth was reached, CAS agar assay was performed and photographed after 3 hours. (B) Growth of Δ*ssoR* expressing operon *pub* under iron-depleted conditions. In both panels, p*pub*, expressing a copy of *pubABC* on plasmid *in trans* for complementation. The operon was under the control of IPTG-inducible promoter P*tac*. (C) Cyt *c* levels in the strains expressing *pubABC* grown to the early stationary phase. IPTG, 0.5 mM. The heme *c* levels of the WT and cyt *c* deficient strain Δ*ccmF* were set to 1 and 0, respectively. In A and B, experiments were independently performed at least four times and representative data were presented. In C, statistics analysis was performed between indicated mutants and the WT: ns, not significant; *. $p < 0.05$; **, $p < 0.01$; ***, $p < 0.001$.

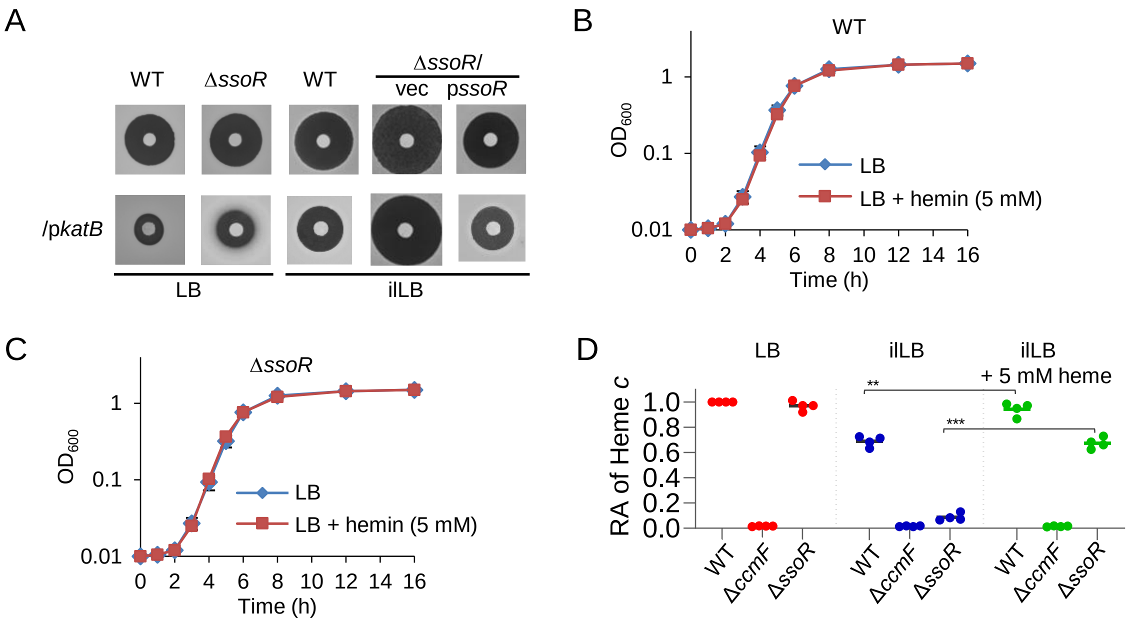

FIG S2. The ssoR mutant has a lowered heme levels. (A) The SsoR loss sensitizes cells grown in ilLB to H_2O_2 revealed by disc diffusion assays. Ten microliters of 30% H_2O_2 was added to filter paper discs (6 mm) on lawns of the indicated strains. Plates were incubated at 30°C for 18 hours. (B-C) Growth of the WT and Δ*ssoR* strains with hemin addition. (D) Cyt *c* levels in the strains under test grown to the early stationary phase. The heme *c* levels of the WT and cyt *c* deficient strain Δ*ccmF* were set to 1 and 0, respectively. In all panels, experiments were independently performed at least four times. In all panels, experiments were independently performed at least four times. In A, representative data were presented, in B-C, the data of four replicates were presented as the mean ± SD, and in D, statistics analysis was performed between indicated samples: ns, not significant; *. $p < 0.05$; **, $p < 0.01$; ***, $p < 0.001$.

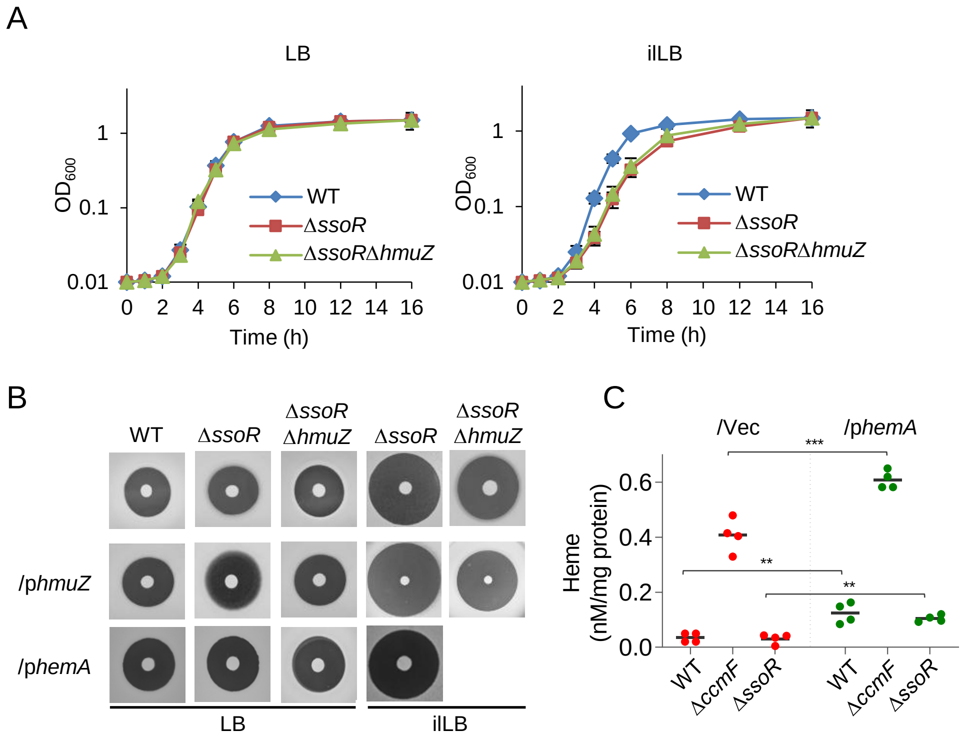

FIG S3. Impacts of heme degradation and biosynthesis on Δ*ssoR*. (A) Growth of the indicated strains in LB and ilLB. (B) H_2O_2 sensitivity of indicated strains. Expression of genes under test was driven by IPTG-inducible promoter P*tac* with IPTG at 0.2 mM. Experiments were carried the same as described in Fig. S2. (C) Intracellular heme concentrations of the relevant strains. In all panels, experiments were independently performed at least four times. In A, the data of four replicates were presented as the mean ± SD. In B, representative data were presented. In C, statistics analysis was performed between indicated samples: ns, not significant; *. $p < 0.05$; **, $p < 0.01$; ***, $p < 0.001$.

A

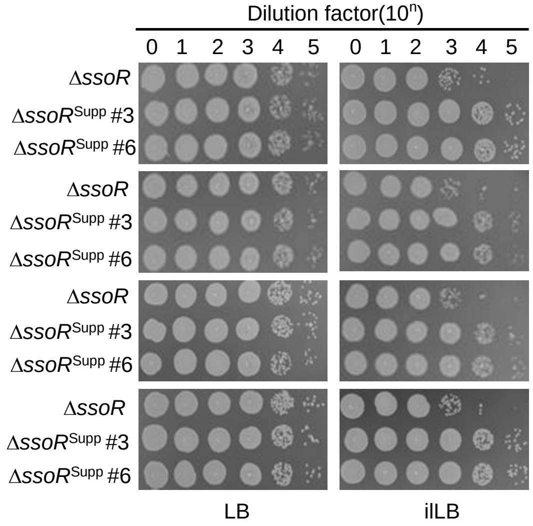

B

Bacterial one-hybrid (B1H) assay of SsoR with *pub* and *fur* promoters

Bait Vector pBXcmT	Target Vector pTRG	Colonies on nonselective plates[a]	Colonies on selective plates[b]	Confirmation[c]	Result Interpretation
/--	/--	188/199/247	0/0/0	--	no bait + no target -- no interaction
/--	/SsoR	223/181/154	0/0/0	--	no bait + SsoR -- no interaction
/P*pub*	/--	143/265/167	0/0/0	--	P*pub* + no target -- no interaction
/P*pub*	/SsoR	221/167/198	189/198/177	187/197/177	P*pub* + SsoR-- strong interaction
/P*fur*	/SsoR	183/143/211	2/0/1	0/0/0	P*fur*+ SsoR -- no interaction

a. M9 agar + 25 µg/ml chloramphenicol + 12.5 µg/ml tetracycline, colonies in three independent experiments.

b. a + 5 mM 3-AT

c. b + 12.5 µg/ml streptomycin

FIG S4. Verification of suppressors and B1H assay. (A) Growth of representative suppressors on plates. (B) Assessment of the interaction between SsoR and the fur promoter with B1H assays. The *pub* promoter was used as the positive control for SsoR.

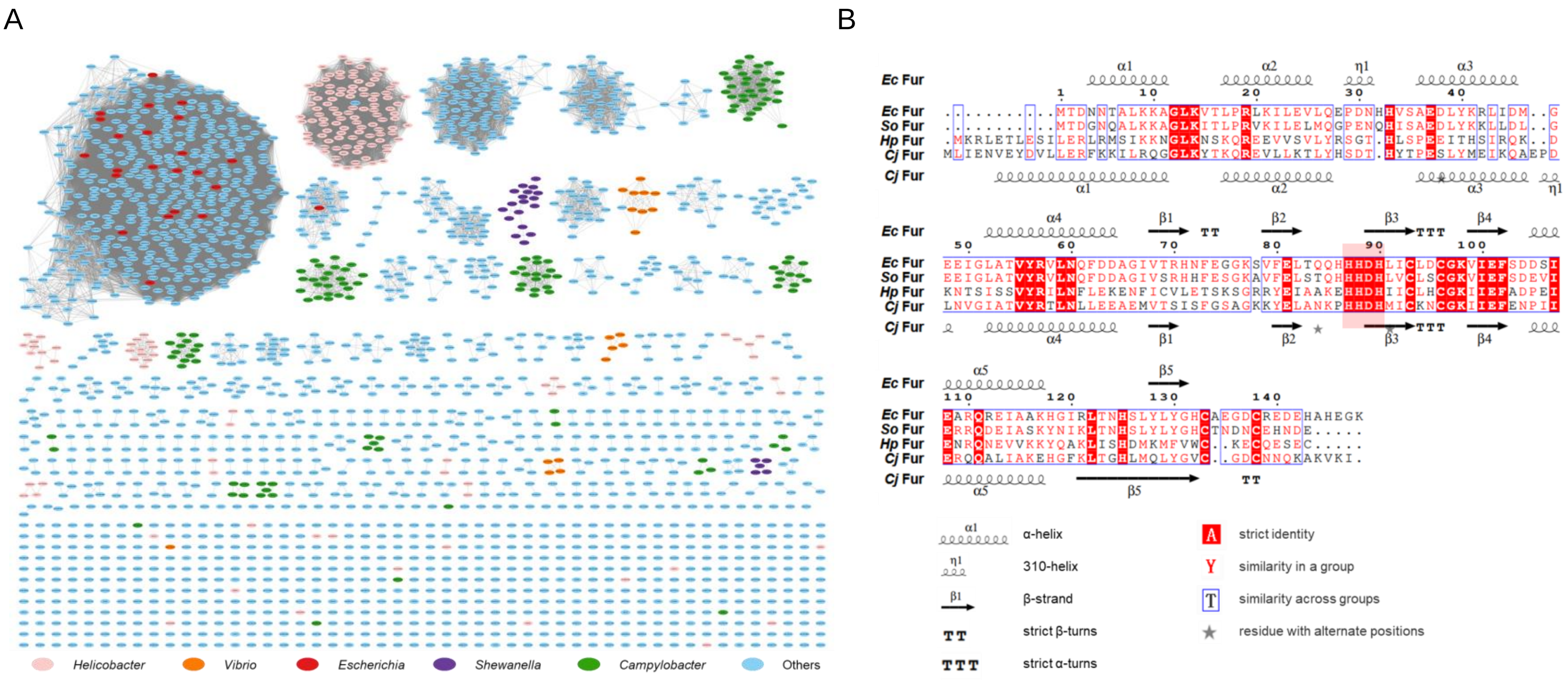

FIG S5. Sequence similarity network (SSN) of Fur homologs. (A) The SSN was generated using EFI-EST based on 2,222 sequences retrieved from UniProt database using BLASTp (E-value cutoff of 1e−5). The resulting network reveals multiple clusters, with each node representing a protein. Nodes are color-coded to highlight five key genera (*Helicobacter*, *Vibrio*, *Escherichia*, *Shewanella*, and *Campylobacter*) that are discussed in the text. Proteins from all other genera are collectively labeled as "Others" and shown in light blue. (B) Amino acid sequence alignment of full length *So*Fur with *Ec*Fur, *Cj*Fur and *Hp*Fur.

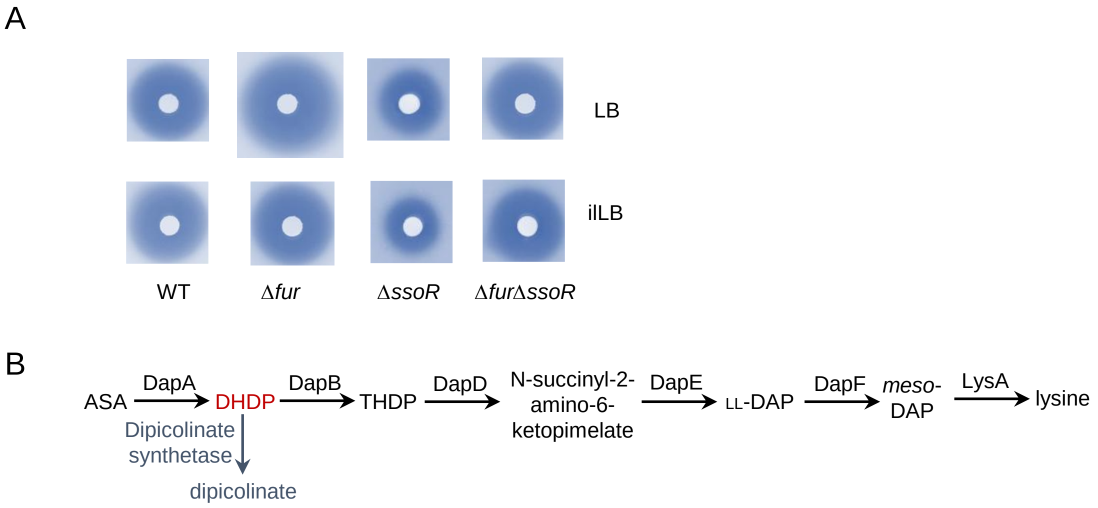

FIG S6. Assessment of iron levels. (A) Free iron levels reflected by susceptibility to streptonigrin (SNG). Ten microliters of 2 mg/ml SNG was added to filter paper discs (6 mm) on cultures of indicated strains. Plates were incubated at 30° C for 24 h. (B) Pathway of diaminopimelate and lysine biosynthesis. The conversion of dihydrodipicolinate (DHDP) to dipicolinate by *B. subtilis* dipicolinate synthase was shown in blue. ASA, aspartate semialdehyde; THDP, tetrahydrodipicolinate, LL-DAP, L,L-diaminopimelate. Experiments were performed at least four times, and representative results were shown.

Table S1 Genes and Proteins significantly different in abundance

Locus	Gene	Annotated function	Transcriptomics		Proteomics	
			log2fc	padjust	log2fc	padjust
SO_0001	*mioC*	FMN-binding protein MioC	-1.256	6.54E-15		
SO_0005	*yidD*	membrane protein insertase YidC	-1.287	9.47E-05	0.471	7.41E-02
SO_0052	*secB*	protein-export chaperone SecB	-1.519	1.17E-27	-0.346	1.22E-01
SO_0053	*gpsA*	NAD(P)H-dependent glycerol-3-phosphate dehydrogenase	-1.513	5.18E-29	-0.192	2.05E-01
SO_0060		DUF4118 domain-containing protein	-1.334	1.75E-02		
SO_0070		ATP-binding cassette domain-containing protein	1.024	1.70E-05	0.629	1.20E-02
SO_0083		4-carboxymuconolactone decarboxylase	1.049	1.31E-02		
SO_0090		protein tyrosine phosphatase family protein	1.353	2.78E-04		
SO_0093		concentrative nucleoside transporter, CNT family	1.479	5.35E-04		
SO_0101	*fdnG*	formate dehydrogenase-N, alpha subunit	-1.923	3.04E-56	-2.833	2.48E-04
SO_0102	*fdnH*	formate dehydrogenase iron-sulfur subunit	-2.272	1.02E-70	-3.808	8.30E-07
SO_0103	*fdnI*	formate dehydrogenase subunit gamma	-2.513	1.17E-93	-4.070	2.52E-02
SO_0104	*fdhE*	FdhE protein	-2.259	5.15E-65		
SO_0105	*selA*	L-seryl-tRNA(Ser) seleniumtransferase	-2.139	4.29E-108	-1.569	1.25E-03
SO_0106	*selB*	selenocysteine-specific elongation factor	-2.122	1.62E-75	-1.470	1.71E-03
SO_0107	*fdhD*	formate dehydrogenase accessory sulfurtransferase FdhD	-2.211	7.66E-89	-2.510	7.64E-04
SO_0108	*yedE*	selenium metabolism membrane protein YedE/FdhT	-3.316	1.84E-160	-2.290	7.92E-03
SO_0109	*yedF*	sulfurtransferase-like selenium metabolism protein YedF	-3.140	4.85E-99		
SO_0111		hypothetical protein	1.028	1.19E-02		
SO_0139	*ftnA*	non-heme ferritin	-6.060	1.70E-194	-3.310	5.17E-04
SO_0147		hypothetical protein	1.101	2.95E-10	0.571	3.54E-03
SO_0154		FUSC family protein	-2.181	1.24E-55	-3.014	1.30E-03
SO_0192		GNAT family N-acetyltransferase	1.087	9.14E-07	0.657	5.17E-03
SO_0197		fatty acid desaturase	-1.003	3.94E-15	-16.610	0.00E+00
SO_0218	*secE*	preprotein translocase subunit SecE	-1.283	4.33E-11	-0.540	6.55E-03
SO_0264	*scyA*	monoheme cytochrome c5 ScyA	-1.708	2.01E-58	-1.645	1.20E-04
SO_0292	*rpe*	ribulose-phosphate 3-epimerase	-1.167	1.44E-18	-0.531	5.27E-02
SO_0312		porin	-2.194	9.20E-24	-2.865	1.33E-03
SO_0313	*potE*	putrescine:ornithine antiporter	-4.312	2.95E-172	-2.666	2.82E-03
SO_0314	*speF*	ornithine decarboxylase	-6.119	0.00E+00	-3.005	2.38E-04
SO_0324		nitrous oxide-stimulated promoter family protein	1.809	5.26E-19	0.435	2.42E-01
SO_0325		DsrE family protein	-1.402	1.15E-12	0.062	6.20E-01
SO_0395	*prmA*	ribosomal protein L11 methyltransferase	-1.280	2.02E-09	0.062	5.07E-01
SO_0397	*frdC*	succinate dehydrogenase subunit C	-1.005	3.87E-09	-0.614	1.39E-01
SO_0398	*frdA*	succinate dehydrogenase flavoprotein subunit	-1.025	6.83E-20	-0.444	5.23E-03
SO_0399	*frdB*	succinate dehydrogenase iron-sulfur subunit	-1.044	8.60E-16	-1.869	3.56E-03
SO_0402		LysR family transcriptional regulator	1.155	1.15E-08	0.020	9.08E-01
SO_0403		hypothetical protein	-1.985	4.21E-15	-0.525	4.37E-02
SO_0404		zinc-dependent metalloprotease	-1.337	5.21E-20	-0.464	8.24E-04
SO_0406	*trxA*	MULTISPECIES: thioredoxin TrxA	-1.392	6.78E-33	-0.236	1.28E-01
SO_0431	*hxpB*	hexitol phosphatase HxpB	-1.195	6.64E-10	-0.272	1.58E-02
SO_0442	*purH*	phosphoribosylaminoimidazolecarboxamide formyltransferase	-1.208	3.02E-08	-0.269	4.93E-03
SO_0444		uncharacterized protein	-1.798	5.15E-50	-1.915	8.88E-03
SO_0452	*trxC*	thioredoxin TrxC	-1.301	1.52E-16	-0.069	6.87E-01
SO_0505		sel1 repeat family protein	1.274	1.45E-17	0.620	1.93E-02
SO_0514		STAS/SEC14 domain-containing protein	-1.121	3.90E-13	0.048	8.04E-01
SO_0526		GNAT family N-acetyltransferase	-1.177	1.39E-05	0.192	3.66E-01
SO_0529		LysR family transcriptional regulator	1.087	2.19E-10	-0.483	1.03E-01
SO_0541		metallo-beta-lactamase family protein	-1.057	3.79E-12	-0.368	1.28E-02
SO_0548		DNA-binding protein HU-beta	-1.297	1.07E-17	-0.153	1.94E-01
SO_0552		hypothetical protein	1.562	4.17E-14		
SO_0556		hypothetical protein	-1.098	3.19E-07	-0.059	8.82E-01
SO_0559		dehydratase	-1.034	2.48E-07	0.017	8.28E-01
SO_0567		1-acyl-sn-glycerol-3-phosphate acyltransferase	-1.099	1.29E-07	0.760	3.03E-03
SO_0572		crotonase/enoyl-CoA hydratase family protein	2.087	7.53E-37		
SO_0581		hypothetical protein	-1.360	3.33E-14	-1.597	4.67E-04
SO_0582		thiopurine S-methyltransferase	-1.425	3.23E-29	-0.196	3.38E-01
SO_0583		bacterioferritin-associated ferredoxin	2.844	1.60E-10		
SO_0598		ADP-dependent NAD(P)H-hydrate dehydratase	-2.011	1.38E-57	-0.754	2.85E-02
SO_0608	*petA*	ubiquinol-cytochrome c reductase iron-sulfur subunit	-1.604	1.47E-17	-0.712	2.54E-03
SO_0645		phage protein	-1.247	1.09E-02		
SO_0648		DUF3164 family protein	-1.273	1.27E-02	0.407	5.10E-01
SO_0649		hypothetical protein	-1.300	1.21E-05	-0.866	1.24E-01
SO_0662		DUF2730 domain-containing protein	-1.798	1.27E-02		
SO_0674		phage protease	-2.060	4.33E-68	-0.768	9.30E-02
SO_0675		Mu-like prophage major head subunit gpT family protein	-2.313	6.90E-66	-1.013	6.40E-03
SO_0676		phage protein	-1.723	5.30E-15	-0.511	2.26E-01
SO_0677		DUF1320 domain-containing protein	-1.843	1.80E-07		
SO_0678		hypothetical protein	-1.915	3.27E-09	-0.837	4.17E-03
SO_0679		hypothetical protein	-1.742	1.50E-25		
SO_0680		tape measure protein	-1.581	1.26E-20		
SO_0682		hypothetical protein	-1.010	2.42E-07		
SO_0703	*groES*	chaperonin GroES	-1.244	3.21E-26		
SO_0716	*sorB*	cytochrome c	1.221	1.70E-02		
SO_0717	*sorD*	c-type cytochrome	1.212	3.47E-02	0.755	3.50E-02

SO_0744	*fbpA*	iron(III) transport system substrate-binding protein	1.319	2.57E-17	0.464	4.61E-03
SO_0779	*gcvT*	glycine cleavage system aminomethyltransferase	-1.226	2.11E-09	0.213	8.26E-02
SO_0797	*traF*	conjugal transfer protein TraF	1.283	2.82E-04		
SO_0798		TonB-dependent receptor	1.524	2.36E-10	-0.064	8.04E-01
SO_0799		cupin domain-containing protein	1.148	6.47E-14	0.572	2.69E-02
SO_0809	*azu*	azurin	-1.920	6.97E-58		
SO_0810	*rbsK*	ribokinase	-2.271	1.57E-37	-1.087	4.17E-03
SO_0811	*rihA*	pyrimidine-specific ribonucleoside hydrolase	-2.747	4.89E-87	-1.511	3.59E-03
SO_0817		LysR family transcriptional regulator, regulator for metE and metH	-1.691	6.19E-14		
SO_0820	*macA*	efflux RND transporter periplasmic adaptor subunit	-1.825	2.32E-20	-16.610	0.00E+00
SO_0821	*macB*	MacB family efflux pump subunit	-1.294	6.55E-11	-2.831	3.66E-04
SO_0822	*macC*	efflux transporter outer membrane subunit	-1.805	7.97E-14	-2.646	1.34E-02
SO_0837	*blaA*	beta-lactamase class D OXA-48	1.884	1.66E-40	-0.164	2.05E-01
SO_0845	*napB*	nitrate reductase (cytochrome), electron transfer subunit	-1.318	1.89E-06	-0.485	9.26E-02
SO_0848	*napA*	nitrate reductase (cytochrome)	-1.432	2.56E-17	-0.882	5.12E-04
SO_0849	*napD*	periplasmic nitrate reductase NapD	-1.257	8.92E-04	-0.264	1.16E-01
SO_0851		prepilin-type N-terminal cleavage/methylation domain-containing protein	-1.752	2.36E-22		
SO_0852		prepilin-type N-terminal cleavage/methylation domain-containing protein	-1.489	1.66E-13		
SO_0853		GspH/FimT family pseudopilin	-1.991	3.97E-39		
SO_0861	*ygfZ*	tRNA-modifying protein YgfZ	-1.087	3.96E-25	0.343	5.41E-02
SO_0862	*serA*	phosphoglycerate dehydrogenase	-1.266	2.81E-21	0.065	6.67E-01
SO_0876	*pepB*	PepB aminopeptidase	-1.250	2.81E-35	-0.356	2.57E-02
SO_0902	*nqrA*	Na+-transporting NADH:ubiquinone oxidoreductase subunit A	1.100	2.52E-13	0.300	6.27E-02
SO_0916		GNAT family N-acetyltransferase	-1.069	6.86E-08	-0.956	2.32E-03
SO_0929		S-adenosylmethionine synthetase	-1.332	2.27E-34	-0.053	1.73E-01
SO_0935	*nhaD*	sodium:proton antiporter NhaD	-1.657	1.30E-23	-1.757	7.28E-04
SO_0994		DUF2846 domain-containing protein	-1.055	1.36E-04	-0.494	3.53E-02
SO_1030	*metH*	methionine synthase	-1.528	1.98E-29	0.258	3.00E-02
SO_1070	*katB*	catalase HPII KatB	-0.162	4.85E-01	-0.162	2.31E-01
SO_1087		Na+/H+ antiporter NhaC family protein	-1.023	9.04E-08		
SO_1091		DMT family transporter	-1.414	9.50E-09		
SO_1094		GreA/GreB family elongation factor	2.566	4.88E-56	6.026	0.00E+00
SO_1103	*nqrA*	Na+-transporting NADH:ubiquinone oxidoreductase subunit A	-1.137	4.34E-06	-0.271	1.54E-02
SO_1111	*brf2*	bacterioferritin	-2.120	3.06E-14	0.609	1.61E-02
SO_1112	*brf1*	bacterioferritin	-1.575	6.48E-13	0.233	5.90E-01
SO_1125		DMT family transporter	-1.227	1.39E-04		
SO_1126	*dnaK*	molecular chaperone DnaK	-1.196	9.38E-18	-0.249	7.82E-03
SO_1159		DNA polymerase III subunit psi	-1.165	6.65E-06		
SO_1162	*lipB*	lipoyl(octanoyl) transferase	-1.062	2.42E-06	0.185	1.67E-02
SO_1175		zinc ribbon-containing protein	-1.155	2.38E-18	0.162	2.43E-01
SO_1182		hypothetical protein	1.055	1.60E-01		
SO_1188		PepSY-associated TM helix domain-containing protein	-3.873	2.74E-50	-1.512	1.11E-02
SO_1189		DUF2271 domain-containing protein	-4.542	1.29E-63	-16.610	0.00E+00
SO_1190		DUF4198 domain-containing protein	-6.034	1.53E-99	-6.466	1.73E-05
SO_1195	*yhbY*	ribosome assembly RNA-binding protein	-1.266	1.77E-07	0.055	5.56E-01
SO_1249		U32 family peptidase	-1.152	6.23E-04	-1.037	1.96E-03
SO_1250		U32 family peptidase	-2.108	1.42E-34		
SO_1252	*yegQ*	tRNA 5-hydroxyuridine modification protein	-1.330	2.73E-07	-0.489	5.57E-03
SO_1258		adenylosuccinate synthase	-1.132	3.71E-15	-0.097	7.57E-02
SO_1284	*rpoD*	RNA polymerase primary sigma factor	-1.044	1.72E-09	-0.359	5.08E-03
SO_1288	*rpsU*	small subunit ribosomal protein S21	-1.056	1.72E-06	-0.575	2.94E-02
SO_1317		hypothetical protein	1.342	1.97E-10	1.507	4.44E-03
SO_1322		adenosylhomocysteine nucleosidase	-1.251	1.04E-07	-0.051	7.79E-01
SO_1324	*gltD*	glutamate synthase (NADPH) small chain	1.454	4.70E-17	1.963	1.32E-03
SO_1325	*gltB*	glutamate synthase (NADPH) large chain	1.998	4.39E-60	1.789	1.26E-03
SO_1336		Na+:H+ antiporter, NhaA family	2.278	3.63E-63		
SO_1381		AhpC/TSA family protein	-1.410	1.84E-02		
SO_1383		DEAD/DEAH box helicase	-1.466	7.99E-07	-0.520	8.40E-03
SO_1388		Xaa-Pro aminopeptidase	-1.136	4.89E-06	-0.368	7.81E-03
SO_1427	*dmsE*	DmsE family decaheme c-type cytochrome	-1.914	2.50E-38	-2.111	8.76E-02
SO_1428	*dmsF*	MtrB/PioB family decaheme-associated outer membrane protein	-1.815	1.59E-23		
SO_1489		hypothetical protein	-1.080	2.32E-15	0.119	9.85E-02
SO_1518	*lldG*	L-lactate dehydrogenase complex protein LldG	-1.386	5.39E-06	-0.585	1.51E-02
SO_1519	*lldF*	L-lactate dehydrogenase complex protein LldF	-1.511	3.27E-37	-1.240	6.31E-04
SO_1520	*lldE*	L-lactate dehydrogenase complex protein LldE	-1.574	1.70E-32	-0.694	2.57E-03
SO_1521	*dld*	D-lactate dehydrogenase	-1.015	6.73E-18	-0.732	5.76E-04
SO_1522		lactate permease	-1.139	1.04E-16	-0.041	8.00E-01
SO_1524	*grpE*	molecular chaperone GrpE	-1.067	8.15E-16	-0.513	7.86E-03
SO_1571		NAD(P)-binding protein	-2.183	6.27E-69	-1.508	9.49E-04
SO_1576		GSH-dependent disulfide-bond oxidoreductase	-1.282	1.07E-18	0.217	2.14E-02
SO_1577		glutathione S-transferase	-1.071	1.73E-06	-16.610	0.00E+00
SO_1581		alkylphosphonate utilization protein	-1.665	7.56E-15	-0.705	4.16E-03
SO_1608	*queF*	7-cyano-7-deazaguanine reductase	-1.091	4.36E-08	-0.045	5.51E-01
SO_1673	*ompW*	outer membrane protein	-3.111	3.13E-88		
SO_1698		MULTISPECIES: DP-EP family protein	-1.974	1.21E-41	1.157	2.10E-01
SO_1738		YajD family HNH nuclease	-1.172	2.94E-10	-0.245	1.24E-01
SO_1746		DUF3624 domain-containing protein	1.327	7.26E-03		
SO_1755		phosphomannomutase	-1.845	6.98E-41	-1.580	1.90E-04
SO_1757		GyrI-like domain-containing protein	-1.165	3.98E-08		

SO_1771		GntP family permease	-1.157	7.73E-10		
SO_1779	*omcA*	decaheme c-type cytochrome OmcA	-1.095	5.49E-20	-0.820	4.30E-02
SO_1782	*mtrD*	DmsE family decaheme c-type cytochrome	1.252	3.27E-03		
SO_1790	*ppiB*	peptidyl-prolyl cis-trans isomerase	-1.318	6.33E-14	-0.169	1.09E-01
SO_1792	*folD*	methylenetetrahydrofolate dehydrogenase (NADP+)	-1.070	6.16E-11	0.286	2.24E-02
SO_1797	*hupB*	DNA-binding protein HU-beta	-1.090	1.00E-15	0.096	1.48E-01
SO_1835		ribosome-associated protein	-1.925	1.30E-11	-0.943	7.21E-03
SO_1836		ribonuclease E inhibitor RraB	-1.851	1.18E-31	-0.841	6.58E-03
SO_1849		hypothetical protein	-1.140	8.77E-07	0.114	4.17E-01
SO_1855	*rmf*	ribosome modulation factor	1.496	5.76E-49	1.798	2.60E-02
SO_1879		4-hydroxy-tetrahydrodipicolinate synthase	-1.030	2.83E-09	0.218	1.38E-01
SO_1881		efflux RND transporter periplasmic adaptor subunit	-1.936	1.34E-22	-0.585	2.18E-03
SO_1921		DUF2986 domain-containing protein	-1.145	2.85E-07	0.526	1.13E-01
SO_1925		efflux RND transporter periplasmic adaptor subunit	1.330	5.71E-11	1.683	3.64E-05
SO_1937	*fur*	ferric uptake regulation protein	2.379	5.86E-18	1.321	1.49E-03
SO_1967		hypothetical protein	-2.150	7.56E-63	-16.610	0.00E+00
SO_1970		hypothetical protein	1.360	2.19E-06	6.026	0.00E+00
SO_1985		ChrR family anti-sigma-E factor	1.056	2.11E-11	0.390	8.59E-02
SO_2005		dksA-type zinc finger protein	-1.076	8.17E-25	-1.326	2.19E-02
SO_2016	*htpG*	molecular chaperone HtpG	-1.246	5.43E-30	-0.436	1.25E-03
SO_2018	*adk*	adenylate kinase	-1.137	2.96E-18	0.143	1.53E-01
SO_2044	*gloA*	lactoylglutathione lyase	-1.116	4.23E-13	0.149	1.94E-01
SO_2045		cation diffusion facilitator family transporter	-1.395	1.38E-22	-0.295	3.24E-01
SO_2046		MarR family transcriptional regulator	-1.178	5.25E-09	0.051	8.21E-01
SO_2090	*hypE*	hydrogenase expression/formation protein HypE	-1.357	1.10E-28	0.391	1.37E-02
SO_2091	*hypD*	hydrogenase expression/formation protein HypD	-1.592	1.93E-49	-1.255	4.84E-03
SO_2111		hypothetical protein	-1.003	1.84E-09	0.279	8.10E-02
SO_2128		hypothetical protein	1.039	2.47E-04		
SO_2174		GGDEF domain protein	1.325	8.26E-33		
SO_2178	*ccpA*	cytochrome c peroxidase	-3.482	4.37E-192	-1.468	3.65E-03
SO_2194	*pdsO*	sortase-associated OmpA-like protein PdsO	4.824	2.52E-48		
SO_2195		marine proteobacterial sortase target protein	3.241	3.08E-48	1.448	2.85E-01
SO_2263	*iscR*	Rrf2 family transcriptional regulator	1.237	1.86E-13	1.358	5.51E-05
SO_2264	*iscS*	IscS subfamily cysteine desulfurase	1.224	1.20E-22	0.509	1.48E-03
SO_2265	*iscU*	nitrogen fixation protein NifU and related proteins	1.918	3.48E-28	0.682	5.59E-03
SO_2266	*iscA*	iron-sulfur cluster assembly protein	1.599	4.23E-12	0.390	3.67E-02
SO_2267	*hscB*	molecular chaperone HscB	1.272	2.24E-09	0.302	8.82E-03
SO_2269	*fdx*	ferredoxin, 2Fe-2S	1.194	1.28E-10	-0.468	3.16E-02
SO_2273		hypothetical protein	-1.599	8.31E-17		
SO_2274	*ndk*	nucleoside-diphosphate kinase [EC:2.7.4.6]	-1.265	6.93E-40	-0.223	4.31E-02
SO_2280		MFS transporter, DHA1 family, multidrug resistance protein	-1.708	1.29E-30		
SO_2297		CPXCG motif-containing cysteine-rich protein	1.093	6.32E-04	0.927	2.06E-01
SO_2303	*trxB*	thioredoxin reductase (NADPH)	-1.067	1.13E-24	-0.070	4.44E-01
SO_2311		hypothetical protein	1.111	8.55E-03		
SO_2324		purine-binding chemotaxis protein CheW	1.111	1.42E-03		
SO_2337	*msrA*	peptide-methionine (S)-S-oxide reductase	-1.210	6.56E-08	0.480	8.93E-02
SO_2342	*nadA*	quinolinate synthase	-1.175	5.00E-08	-0.351	4.17E-02
SO_2354	*ttcA*	tRNA 2-thiocytidine biosynthesis protein TtcA	-1.507	1.04E-08		
SO_2355	*uspE*	universal stress protein E	-1.477	1.95E-28	0.051	5.15E-01
SO_2364	*ccoN*	cytochrome c oxidase cbb3-type subunit I	-1.260	1.00E-42	-0.407	1.41E-02
SO_2387		DTW domain-containing protein	-1.012	1.53E-03		
SO_2389	*emrD*	multidrug efflux MFS transporter EmrD	-1.393	2.83E-07		
SO_2407		YjjI family glycine radical enzyme	4.158	0.00E+00	4.884	1.07E-04
SO_2408		YjjW family glycine radical enzyme activase	4.755	4.43E-208	6.026	0.00E+00
SO_2413	*ubiG*	bifunctional 3-demethylubiquinone 3-O-methyltransferase	-1.145	1.55E-14	-0.269	8.92E-03
SO_2426	*ssoR*	two-component system, OmpR family, response regulator	-13.787	1.72E-23	-3.144	8.63E-02
SO_2446		hypothetical protein	1.066	7.53E-09	0.447	5.75E-02
SO_2476	*kdsB*	3-deoxy-manno-octulosonate cytidylyltransferase	-1.202	1.52E-10		
SO_2501	*queE*	7-carboxy-7-deazaguanine synthase	-1.107	1.16E-04	-0.432	3.53E-02
SO_2509	*rsxB*	electron transport complex subunit RsxB	-1.079	7.53E-12	-0.499	5.59E-03
SO_2585		hypothetical protein	1.098	1.76E-21		
SO_2610		YchF/TatD family DNA exonuclease	-1.026	3.67E-04	-0.222	1.25E-01
SO_2618	*apbC*	iron-sulfur cluster carrier protein ApbC	-1.082	6.83E-15	0.033	7.65E-01
SO_2639		hypothetical protein	1.619	8.92E-13		
SO_2647		cytosolic long-chain acyl-CoA thioester hydrolase family protein	-1.536	2.24E-04	-0.503	2.91E-02
SO_2659		Mu phage protein Kil	-1.090	8.91E-03		
SO_2684		Mu phage protease GpI	-2.876	5.20E-40	-0.080	6.40E-01
SO_2685		Mu-like prophage major head subunit gpT family protein	-1.933	4.76E-18		
SO_2686		phage protein	-1.780	2.30E-02		
SO_2687		phage protein	-1.688	1.43E-03		
SO_2688		phage protein	-1.406	1.13E-09		
SO_2690		phage virion morphogenesis protein	-1.567	2.90E-02		
SO_2691		hypothetical protein	-1.515	1.37E-02		
SO_2711		hypothetical protein	1.028	1.55E-17	0.491	4.20E-02
SO_2726		cytochrome b/b6 domain-containing protein	-1.337	4.04E-16	-16.610	0.00E+00
SO_2730	*pepE*	dipeptidase E	-1.084	8.38E-15	0.112	1.87E-01
SO_2737	*bioD*	dethiobiotin synthase	2.474	1.21E-49	1.296	1.96E-03
SO_2738	*bioC*	malonyl-ACP O-methyltransferase BioC	2.657	4.38E-100	1.508	2.25E-03
SO_2739	*bioF*	8-amino-7-oxononanoate synthase	3.317	2.01E-82	1.535	2.63E-03

SO_2740	*bioB*	biotin synthase BioB	2.239	4.13E-44	0.790	1.32E-03
SO_2759	*upp*	uracil phosphoribosyltransferase	-1.231	1.51E-24	0.136	1.98E-01
SO_2760	*purM*	phosphoribosylformylglycinamidine cyclo-ligase	-1.443	2.96E-12	-0.320	1.28E-01
SO_2775	*acpP*	acyl carrier protein	-1.811	1.36E-12	1.121	1.65E-01
SO_2834	*nrdD*	ribonucleoside-triphosphate reductase (formate)	-1.360	3.26E-35	-0.879	3.60E-03
SO_2841		hypothetical protein	2.441	1.90E-28		
SO_2849		hypothetical protein	1.018	1.34E-04	1.435	1.06E-03
SO_2915	*ackA*	acetate kinase	-1.029	1.12E-09	0.498	5.16E-03
SO_2918		hypothetical protein	-1.399	1.06E-14	6.026	0.00E+00
SO_2921	*folX*	dihydroneopterin triphosphate 2\\'-epimerase	-1.063	1.23E-05	-0.321	4.20E-03
SO_2923	*gltS*	glutamate:Na+ symporter, ESS family	-1.125	2.53E-11		
SO_2929		hypothetical protein	-1.013	6.67E-16	0.058	5.66E-01
SO_2940		DUF1983 domain-containing protein	-1.519	5.49E-39	-1.619	4.36E-04
SO_2941		tail assembly protein	-1.973	9.54E-18	-1.336	1.08E-03
SO_2945		Lambda phage tail fiber protein	-2.124	7.29E-51	-16.610	0.00E+00
SO_2946		hypothetical protein	-2.422	9.03E-21	0.000	1.00E+00
SO_2947		Lambda phage protein of known function	-2.097	7.48E-10		
SO_2948		C40 family peptidase	-1.529	1.01E-10	-1.226	3.55E-02
SO_2949		phage minor tail protein L	-1.181	4.20E-12		
SO_2950		hypothetical protein	-1.253	6.00E-10	-0.647	8.02E-02
SO_2952		phage tail protein	-1.695	9.55E-11	-16.610	0.00E+00
SO_2953		phage tail tape measure protein	-1.969	1.52E-46	-16.610	0.00E+00
SO_2955		phage tail assembly chaperone family protein, TAC	-2.073	1.17E-47	-1.104	1.11E-01
SO_2956		tail protein	-2.090	2.62E-71	-0.797	8.81E-03
SO_2957		DUF3168 domain-containing protein	-1.876	1.29E-15		
SO_2958		HK97 gp10 family phage protein	-1.805	1.34E-11		
SO_2960		phage head closure protein	-1.738	5.64E-07		
SO_2961		head-tail connector protein	-1.898	4.34E-07		
SO_2962		hypothetical protein	-1.677	6.57E-11	-0.998	1.38E-02
SO_2963		phage major capsid protein	-2.186	6.94E-66	-1.109	1.37E-03
SO_2964		Clp protease ClpP	-1.966	1.41E-31	-0.690	1.26E-03
SO_2965		phage portal protein	-1.934	1.50E-37	-0.177	5.09E-01
SO_2969		HNH endonuclease	-1.486	2.08E-09		
SO_2970		phage protein	-1.714	5.00E-14		
SO_2971		hypothetical protein	-1.795	3.88E-15		
SO_2972		hypothetical protein	-1.840	7.45E-18		
SO_2973		lysozyme	-2.641	6.70E-21		
SO_2974		Lambda phage pyridoxal phosphate dependent enzyme	-3.007	6.00E-09	-16.610	0.00E+00
SO_2990		helix-turn-helix transcriptional regulator	-1.352	1.16E-17	-0.064	7.03E-01
SO_3009		hypothetical protein	-1.250	3.33E-02		
SO_3025		alpha/beta hydrolase	-3.067	5.64E-123	-6.870	1.31E-03
SO_3029		DUF2798 domain-containing protein	-1.289	1.14E-05		
SO_3030	*pubA*	SidA/IucD/PvdA family monooxygenase	-4.323	7.31E-106	-2.933	8.48E-06
SO_3031	*pubB*	acetyltransferase	-4.359	2.02E-159	-3.555	1.01E-03
SO_3032	*pubC*	IucA/IucC family siderophore biosynthesis protein	-4.565	1.18E-275	-3.144	5.10E-06
SO_3033	*putA*	TonB-dependent siderophore receptor	-4.315	0.00E+00	-2.931	5.33E-03
SO_3034	*putB*	siderophore-iron reductase	-3.779	3.16E-115	-4.154	1.93E-04
SO_3054		MBL fold metallo-hydrolase	-1.248	1.39E-14	-0.614	1.36E-02
SO_3062		hypothetical protein	-1.959	2.40E-14		
SO_3063		alanine:cation symporter family protein	-1.061	6.83E-10	-2.817	8.08E-02
SO_3084		EAL domain-containing protein	-1.184	8.66E-26	-0.946	7.61E-02
SO_3086		fatty acid oxidation complex subunit alpha FadJ	2.328	3.01E-55		
SO_3099	*fadL*	long-chain fatty acid transport protein	-1.230	2.42E-13	-0.686	7.16E-03
SO_3140		thymidine kinase	-1.065	9.92E-13	-0.017	7.72E-01
SO_3194	*rfaH*	transcriptional antiterminator RfaH	-1.099	6.72E-09	-16.610	0.00E+00
SO_3229	*fliE*	flagellar hook-basal body complex protein FliE	1.111	2.16E-10		
SO_3282		methyl-accepting chemotaxis protein	-1.489	1.60E-32	-0.513	5.17E-03
SO_3286	*cydA*	cytochrome bd ubiquinol oxidase subunit I	-1.388	4.21E-15	-0.067	4.50E-01
SO_3293	*guaB*	IMP dehydrogenase	-1.102	5.14E-21	-0.352	1.62E-03
SO_3315	*rlmN*	23S rRNA (adenine2503-C2)-methyltransferase	-1.144	7.43E-10	-0.208	1.55E-01
SO_3319		DoxX family protein	2.733	7.05E-41		
SO_3325		hypothetical protein	-1.126	1.45E-07	-0.264	3.52E-02
SO_3344		hypothetical protein	2.113	6.47E-06		
SO_3367	*trmB*	tRNA (guanine-N7-)-methyltransferase	-1.308	1.29E-07	-0.132	3.69E-01
SO_3370		YceI family protein	2.108	8.05E-76	1.546	4.11E-04
SO_3371	*cybB*	cytochrome b	1.970	3.32E-30		
SO_3376		DUF3833 domain-containing protein	1.030	6.95E-07		
SO_3377		chalcone isomerase family protein	1.088	3.85E-14	0.654	4.27E-01
SO_3380		DUF1365 domain-containing protein	1.026	1.04E-09	1.041	8.08E-02
SO_3403	*raiA*	ribosome-associated translation inhibitor RaiA	-1.805	3.95E-34	-0.431	4.98E-02
SO_3406		DUF3649 domain-containing protein	2.553	3.11E-23	6.026	0.00E+00
SO_3407		PepSY domain-containing protein	1.427	1.15E-08	0.557	1.06E-03
SO_3410		hypothetical protein	1.101	6.23E-24		
SO_3420		cytochrome c	-1.648	1.39E-46	-0.793	5.57E-03
SO_3421		cytochrome b/b6 domain-containing protein	-1.788	3.91E-51	-3.402	2.50E-02
SO_3423		DNA polymerase III subunit chi	-1.385	1.21E-09	0.492	4.29E-02
SO_3439	*ftsB*	cell division protein FtsB	-1.254	1.78E-03		
SO_3488		helix-turn-helix transcriptional regulator	1.089	2.89E-12		
SO_3494	*mexR*	TetR/AcrR family transcriptional regulator	-1.011	2.28E-08	0.286	5.70E-02

SO_3496	*puuC*	aldehyde dehydrogenase	-1.206	1.99E-22	-0.278	3.98E-02
SO_3504		DUF5009 domain-containing protein	-1.073	2.63E-04		
SO_3505	*nagA*	N-acetylglucosamine-6-phosphate deacetylase	-1.090	1.05E-07	0.380	1.35E-02
SO_3506		SIS domain-containing protein	-1.140	1.02E-08	0.023	9.10E-01
SO_3554	*purE*	5-(carboxyamino)imidazole ribonucleotide mutase	-1.307	3.02E-06	-0.197	1.66E-01
SO_3583		16S rRNA pseudouridine516 synthase [EC:5.4.99.19]	1.641	1.28E-27		
SO_3585		NAD(P)H-dependent oxidoreductase	3.630	1.20E-48	6.026	0.00E+00
SO_3586		VOC family protein	2.607	1.87E-56	2.310	6.04E-04
SO_3596		DUF1289 domain-containing protein	1.128	9.60E-09		
SO_3599	*cysP*	sulfate/thiosulfate transport system substrate-binding protein	1.303	4.35E-18	-0.654	1.11E-02
SO_3613	*purT*	formate-dependent phosphoribosylglycinamide formyltransferase	-1.087	1.23E-13	-0.155	2.32E-02
SO_3652	*rplU*	large subunit ribosomal protein L21	-1.155	1.97E-07	-0.423	5.92E-02
SO_3668	*hmuX*	heme iron utilization protein	1.211	1.32E-07	0.546	2.85E-03
SO_3669	*hmuA*	hemoglobin/transferrin/lactoferrin receptor protein	2.527	1.60E-08	0.498	3.91E-03
SO_3670	*tonB*	periplasmic protein TonB	3.421	3.49E-13	2.336	2.63E-03
SO_3671	*exbB*	biopolymer transport protein ExbB	2.148	2.53E-20	0.779	3.65E-03
SO_3672	*exbD*	biopolymer transporter ExbD	1.712	1.80E-18	0.707	9.24E-02
SO_3695	*pyrC*	dihydroorotase	-1.472	2.57E-07	-0.240	8.61E-03
SO_3705		adenosylhomocysteine nucleosidase	-2.605	7.12E-23	-1.085	1.50E-04
SO_3709	*btuF*	vitamin B12 transport system substrate-binding protein	1.035	1.74E-10	0.492	1.65E-02
SO_3738	*cysJ*	sulfite reductase (NADPH) flavoprotein alpha-component	1.075	5.56E-07	-0.627	4.87E-02
SO_3764		DUF2170 family protein	2.197	2.85E-67	1.136	3.53E-02
SO_3765		PspA/IM30 family protein	1.163	2.22E-14	0.103	7.12E-02
SO_3783		ATP-dependent RNA helicase	-1.076	2.80E-05	-1.005	9.95E-03
SO_3787		hypothetical protein	1.146	1.90E-28	0.238	3.08E-01
SO_3802		ABC-2 type transport system ATP-binding protein	-1.087	1.45E-14	-0.317	7.25E-02
SO_3815		cyclic-di-GMP-binding protein	-1.017	5.38E-15	0.020	6.71E-01
SO_3837		ribose-phosphate pyrophosphokinase	-1.178	1.19E-08	-0.218	2.52E-02
SO_3847		SMI1/KNR4 family protein	-1.028	6.07E-04	-0.130	3.75E-01
SO_3852		MULTISPECIES: DUF1456 family protein	-1.066	1.16E-05	-0.091	5.09E-01
SO_3863	*modA*	molybdate transport system substrate-binding protein	-1.490	2.01E-25	0.282	1.65E-02
SO_3864	*modB*	molybdate transport system permease protein	-1.016	2.77E-18		
SO_3870		disulfide bond formation protein B	1.156	3.54E-22		
SO_3891		DUF3149 domain-containing protein	-2.311	1.40E-66		
SO_3896		porin	-1.460	1.79E-22	-0.459	1.12E-01
SO_3910		hypothetical protein	1.292	1.48E-07		
SO_3913	*piuC*	PKHD-type hydroxylase	1.204	1.04E-12	0.252	7.45E-03
SO_3914		catecholate siderophore receptor	2.863	2.79E-35	0.136	1.73E-01
SO_3915	*cheX*	chemotaxis protein CheX	1.449	1.40E-14	0.781	1.96E-02
SO_3938		uncharacterized protein	1.095	1.23E-03	0.374	2.15E-01
SO_3965		phosphocarrier protein NPr	1.051	3.15E-06		
SO_3980	*nrfA*	nitrite reductase (cytochrome c-552)	-1.123	7.78E-09	-0.506	3.07E-02
SO_4053		methyl-accepting chemotaxis protein	-1.206	4.16E-14	-0.255	2.94E-02
SO_4054	*metF*	MULTISPECIES: methylenetetrahydrofolate reductase	-1.986	3.31E-30	0.149	1.86E-01
SO_4055	*metL*	bifunctional aspartate kinase/homoserine dehydrogenase II	-1.951	1.87E-26	0.646	1.34E-02
SO_4056	*metB*	cystathionine gamma-synthase	-2.083	8.86E-20	0.180	4.13E-01
SO_4060	*phsC*	polysulfide reductase chain C	-1.637	1.24E-05		
SO_4061	*phsB*	polysulfide reductase chain B	-2.874	5.45E-15		
SO_4072	*rimO*	ribosomal protein S12 methylthiotransferase	-1.194	8.34E-05	-0.609	3.32E-03
SO_4118	*maeB*	malate dehydrogenase	-1.031	1.74E-06	-0.405	1.04E-02
SO_4120	*rpmE*	large subunit ribosomal protein L31	-1.062	1.00E-06	-0.093	4.85E-01
SO_4131		NfeD family protein	-1.103	1.08E-04	-0.803	9.18E-02
SO_4162	*hslV*	MULTISPECIES: ATP-dependent protease subunit HslV	-1.299	2.63E-11	-0.736	4.84E-03
SO_4163	*hslU*	ATP-dependent protease ATPase subunit HslU	-1.548	7.16E-34	-0.471	2.83E-03
SO_4164		MULTISPECIES: DUF971 domain-containing protein	-1.022	4.40E-07	0.111	2.49E-01
SO_4169		(6-4)DNA photolyase [EC:4.1.99.13]	1.053	1.01E-04		
SO_4170		SDR family oxidoreductase	1.721	5.37E-10	2.132	8.84E-04
SO_4174		dTDP-4-dehydrorhamnose reductase [EC:1.1.1.133]	-1.005	1.38E-05	0.193	1.64E-01
SO_4196		MULTISPECIES: YgjV family protein	1.142	1.42E-07		
SO_4208	*hemB*	porphobilinogen synthase [EC:4.2.1.24]	-1.149	4.63E-18	-0.002	9.72E-01
SO_4256	*rph*	ribonuclease PH	-1.231	4.78E-10	-0.130	3.91E-01
SO_4257		YicC family protein	-1.026	9.21E-05	-0.190	1.48E-02
SO_4302		iron-sulfur cluster repair di-iron protein	1.453	2.00E-02		
SO_4320	*aggA*	type I protein secretion system protein AggA	-1.244	2.31E-22	0.213	2.67E-02
SO_4323		EAL domain-containing protein	-1.060	1.03E-17	0.302	1.34E-02
SO_4326	*vexR*	TetR/AcrR family transcriptional regulator	1.012	3.77E-11	0.064	7.74E-01
SO_4334	*creD*	cell envelope integrity protein CreD	1.387	2.13E-16	-0.324	4.06E-01
SO_4396	*azoR*	FMN-dependent NADH-azoreductase	1.291	2.12E-34	0.669	2.99E-03
SO_4407		MULTISPECIES: translational GTPase TypA	-1.097	3.40E-13	-16.610	0.00E+00
SO_4410	*glnA*	glutamine synthetase	-1.648	1.10E-18	-0.194	8.08E-02
SO_4422		iron complex outermembrane recepter protein	1.020	6.92E-24		
SO_4461		hypothetical protein	1.058	2.44E-06		
SO_4476	*cpxP*	periplasmic protein CpxP/Spy	-1.782	7.65E-14		
SO_4477	*cpxR*	two-component system, OmpR family, response regulator CpxR	-2.234	1.44E-55	-1.350	3.13E-04
SO_4478	*cpxA*	sensor histidine kinase CpxA	-1.598	1.11E-23	-1.539	1.41E-04
SO_4484		DUF1924 domain-containing protein	1.835	5.04E-07		
SO_4485		diheme cytochrome c	1.119	6.53E-10		
SO_4512	*fdhX*	win-arginine translocation signal domain-containing protein	-2.744	5.09E-26	-1.101	1.48E-01
SO_4513	*fdhA*	formate dehydrogenase major subunit	-1.909	5.10E-32	-4.191	3.76E-04

SO_4514	*fdhB*	formate dehydrogenase iron-sulfur subunit	-1.008	1.19E-04	-3.019	7.17E-03
SO_4515	*fdhC*	formate dehydrogenase subunit gamma	-1.522	4.30E-23	-1.559	4.86E-02
SO_4516		TonB-dependent receptor	-2.657	8.25E-48	-1.219	7.51E-05
SO_4523	*irgA*	outer membrane receptor for ferrienterochelin and colicins	2.815	9.15E-32	0.741	4.55E-04
SO_4524		LysR family transcriptional regulator	2.639	1.89E-32	0.683	1.87E-03
SO_4558		hypothetical protein	-3.991	5.27E-251	-16.610	0.00E+00
SO_4571		LysR family transcriptional regulator	1.162	1.30E-05	0.447	2.03E-02
SO_4590		cysteine hydrolase	-1.005	4.32E-07	0.218	4.82E-01
SO_4592		hypothetical protein	1.447	2.43E-08		
SO_4593		DUF2057 family protein	1.271	2.88E-08		
SO_4606	*coxB*	cytochrome c oxidase subunit II	1.207	1.08E-28		
SO_4611		SURF1 family protein	1.078	5.42E-09		
SO_4618		S9 family peptidase	-2.090	1.34E-45	-1.965	3.62E-05
SO_4639		NirD/YgiW/YdeI family stress tolerance protein	1.019	9.23E-04		
SO_4640		glutaredoxin/glutathione-dependent peroxiredoxin	1.303	3.63E-23	1.627	6.56E-04
SO_4645		choice-of-anchor H family protein	1.779	1.89E-21	6.026	0.00E+00
SO_4651		YezD family protein	1.954	1.42E-12		
SO_4652	*sbp*	sulfate/thiosulfate transport system substrate-binding protein	1.339	2.77E-04		
SO_4666	*cytcB*	cytochrome c4	-1.113	6.10E-13	-0.846	1.49E-02
SO_4674		glycine C-acetyltransferase	-1.135	3.00E-10	-0.090	3.01E-01
SO_4692		efflux RND transporter permease subunit	-1.005	1.71E-11	-1.200	7.90E-04
SO_4693		efflux RND transporter periplasmic adaptor subunit	-1.082	1.50E-25	-0.966	3.23E-04
SO_4699	*prlC*	oligopeptidase A	-1.495	1.09E-51	-0.505	2.01E-03
SO_4700		hypothetical protein	2.096	1.21E-04		
SO_4701		hypothetical protein	1.522	2.59E-13		
SO_4716	*ryhB*	antisense RNA ryhB	1.295	4.20E-08	-0.763	1.15E-03
SO_4740		DUF2061 domain-containing protein	3.516	5.27E-35		
SO_4742	*glmR*	DeoR family transcriptional regulator	-1.333	1.08E-09	0.072	7.05E-01
SO_4743		iron complex outermembrane recepter protein	-3.569	1.66E-57	-4.920	8.26E-05
SO_4761		hypothetical protein	-4.282	6.44E-15		
SO_4819		hypothetical protein	3.272	4.57E-14		
SO_A0072		MULTISPECIES: type II toxin-antitoxin system HigB family toxin	1.121	3.00E-10	0.081	8.77E-01
SO_A0078		MULTISPECIES: type II toxin-antitoxin system ParD family antitoxin	1.155	4.83E-12	0.085	6.99E-01
SO_A0079		type II toxin-antitoxin system RelE/ParE family toxin	1.084	7.61E-19	-0.077	6.65E-01
SO_A0095	*parA*	chromosome partitioning protein	2.668	6.26E-102	-0.089	3.77E-01
SO_A0109		EAL domain-containing protein	1.194	9.06E-06		
SO_A0171		recombinase family protein	1.085	2.38E-08	-0.334	1.76E-02
SO_A0172		recombinase family protein	1.140	6.02E-16	-0.064	8.17E-01

Table S2. Strains and plasmids used in this study

Strain or plasmid	Description	Source/Reference
E. coli strains		
DH5α	Host for cloning	Laboratory stock
WM3064	Δ*dapA*, donor strain for conjugation	W.Metcalf, UIUC
S. oneidensis strains		
MR-1	Wild type	ATCC 700550
HG2426	Δ*ssoR* derived from MR-1	15
HG2889	Δ*ccmF* derived from MR-1	27
HG3030-2	Δ*pub* mutant derived from MR-1	23
HG1937	Δ*fur* mutant derived from MR-1	52
HG3162	Δ*katB* derived from MR-1	33
HG3894	Δ*hmuZ* derived from MR-1	25
HG2426-	Δ*ssoR*Δ*hmuZ* derived from MR-1	This study
HG2426-1937	Δ*ssoR*Δ*fur* derived from MR-1	This study
Plasmids		
pHGM01	Att-based suicide vector, Ap^r, Gm^r, Cm^r	27
pHGEI01	Km^r, integrative *lacZ* reporter vector	58
pHGEN-Ptac	Km^r, IPTG-inducible expression vector	54
pBBR-Cre	Spr, helper plasmid used with pHGEI01	59
pHGT01	Gm^r, vector containing transposable sequence	57
pHGEN-P*tac-ssoR*	Vector for inducible expression of *ssoR*	15
pHGEN-P*tac-pub*	Vector for inducible expression of *pubABC*	15
pHGEN-P*tac-katB*	Vector for inducible expression of *katB*	33
pHGEN-P*tac-hmuZ*	Vector for inducible expression of *hmuZ*	25
pHGEN-P*tac-hemA*	Vector for inducible expression of *hemA*	25
pHGEN-P*tac-BsdpaAB*	Vector for inducible expression of *B. subtilis dpaAB*	25
pHGEN-P*tac-fur*	Vector for inducible expression of *fur*	This study
pHGEN-P*tac-fur*^{M}	Vector for inducible expression of $Fur^{H88L/H90L}$	This study
